# Racial disparities, comorbidities, and low body mass index reduce survival after cardiopulmonary resuscitation: a systematic review and meta-analysis

**DOI:** 10.3389/fpubh.2025.1663865

**Published:** 2025-12-10

**Authors:** Zifan Du, Shuai Ma, Yali Tong, Pengfei Zhao, Zetao Chen, Haojun Fan, Shike Hou, Bin Fan

**Affiliations:** 1 School of Disaster and Emergency Medicine, Tianjin University, Tianjin, China; 2 Wenzhou Safety (Emergency) Institute of Tianjin University, Wenzhou, China

**Keywords:** cardiopulmonary resuscitation, survival rate, ethnicity, past medical history, body mass index, meta-analysis, individualized factors

## Abstract

**Introduction:**

CPR is crucial for the management of cardiac arrest. However, the impacts of certain individualized factors, such as different ethnicities, body weights, and medical histories, on the efficacy of CPR remain unclear. This meta-analysis clarifies the associations between three individualized factors and the outcomes of CPR, aiming to optimize resuscitation strategies.

**Methods:**

We systematically searched eight databases—PubMed, Web of Science, Scopus, Embase, Cochrane Library, VIP, Wan Fang, and CNKI—for studies that explored the associations between ethnicity, past medical history, body mass index (BMI), and CPR outcomes. Separate meta-analyses were then conducted for each of these three individualized factors.

**Results:**

Eleven studies evaluated ethnicity, nine assessed medical history, and eight analyzed BMI. White patients exhibited significantly higher survival rates than Black patients (OR = 1.36, 95% CI [1.22–1.50], *p* < 0.00001). Compared to patients with a medical history, patients without a medical history have a higher survival rate (OR = 0.51, 95% CI [0.37–0.70], *p* < 0.0001). Compared to standard weight groups (BMI 18.5–24.9 kg/m^2^), underweight individuals (BMI < 18.5 kg/m^2^) had lower survival (OR = 0.64, 95%CI[0.51–0.80], *p* = 0.0001) and poorer neurological outcomes in underweight individuals (BMI < 18.5 kg/m^2^) (OR = 0.72, 95%CI[0.55–0.94], *p* = 0.01). No significant differences were observed in overweight/obese versus normal-weight patients.

**Discussion:**

This study demonstrates that ethnicity differences, pre-existing comorbidities, and low BMI can affect survival rates after CPR. These results are of great significance for clinical practice, suggesting that it is necessary to reduce inequalities in the distribution of medical resources in response to racial differences, optimize disease management for patients with comorbidities, and incorporate underweight status into high-risk assessment. Future research should further explore the underlying mechanisms and expand to more regions, so as to provide evidence for the construction of a personalized resuscitation medicine system and the formulation of guidelines.

**Systematic Review Registration:**

https://www.crd.york.ac.uk/PROSPERO/view/CRD42025558162, identifier PROSPERO (CRD42025558162).

## 1 Introduction

Cardiac arrest (CA) is a sudden, life-threatening acute medical event that affects more than 1 million people worldwide each year (1). As a core intervention in the management of CA, the quality of CPR directly determines survival and neurological outcomes. Despite decades of development in CPR techniques and guidelines, survival to hospital discharge for patients with out-of-hospital cardiac arrest (OHCA) remains generally below 10% (2), and the proportion of survivors achieving good neurological recovery is low (3, 4). This stark reality highlights the need to optimize resuscitation. Current standard resuscitation guidelines emphasize the quality of compressions and defibrillation (5, 6). Still, individual patient variability is an issue that cannot be ignored, suggesting the limitations of a “one-size-fits-all” strategy and the need for individualized interventions.

Existing studies have revealed that CPR outcomes may be influenced by multiple individual factors such as race, body composition, and co-morbidity burden, but the specific mechanisms have not been fully elucidated. In terms of racial disparities, several large-scale studies have demonstrated that ethnic minorities (e.g., black people and Latinos) are significantly less likely than white patients to receive bystander CPR in the event of an OHCA in a public place or at home (7, 8). Also, black patients have lower survival rates, with black women having the lowest survival benefit after receiving CPR (9). A study by Margo et al (10) noted that racial disparities in survival from in-hospital CA (IHCA) may be related to the level of nurse staffing in the hospitals in which the patients are admitted, and that the survival benefit of improved hospital staffing may be particularly significant for black patients. Notably, most current research on racial factors is based on U.S. data, and there is a relative lack of analysis of native populations such as African-Americans and Asians. In terms of co-morbidity burden, although the European Resuscitation Council 2021 has issued guidelines for the management of CA in patients with specific comorbidities (11), most studies in the literature on the specific mechanisms by which comorbidities affect CPR outcomes remain at the speculative level. In terms of body weight, the “obesity paradox” associated with CPR remains controversial (12, 13), with some studies suggesting that body weight is negatively correlated with the rate of return of spontaneous circulation (ROSC) in patients with OHCA (14). In contrast, others suggest (15)that subcutaneous fat in obese patients may buffer the impact of chest compressions and reduce the risk of rib fracture, and that lipocalin secreted by visceral fat may inhibit excessive inflammatory responses and provide additional energy reserves, thus providing a potential protective effect.

To explore in depth the influence of the above individualized factors (race, co-morbidity burden, body weight) on CPR outcomes, the present study targeted a meta-analysis. We stratified and parsed the independent effects of these factors by integrating data from multiple sources, including non-English databases, to mitigate geographic bias. Our study aims to advance precision resuscitation guidelines and transition from “one-size-fits-all” CPR to tailored interventions that optimize survival and neurological prognosis.

## 2 Methods

### 2.1 Protocol and registration

This systematic review was conducted by the MOOSE and PRISMA guidelines (16, 17) and was prospectively registered with PROSPERO (CRD42025558162).

### 2.2 Eligibility criteria

We employed the PICO framework for study selection. The population (P) comprised adult patients (≥18 years) receiving CPR. The intervention factors (I) included three predefined personalization parameters:

1. Ethnicity: Per the classification reported in the included studies, the included studies must explicitly document the racial or ethnic classification of participants.
2. Comorbidities: Comorbidities were defined based on patients’ medical records reported in the included studies and diagnosed per the International Classification of Diseases, 10th Revision (ICD-10) coding standards;
3. Body mass index: Participants were strictly stratified according to the WHO grading criteria into four categories: underweight (<18.5 kg/m^2^), normal weight (18.5–24.9 kg/m^2^), overweight (25.0–29.9 kg/m^2^), and obese (30–34.9 kg/m^2^).

The primary outcome (O) was survival to hospital discharge; secondary outcomes included the return of spontaneous circulation (ROSC), survival to hospital admission, and favorable neurological outcome (CPC 1–2 or mRS 0–2).

Exclusion criteria included: pediatric, animal, or human model studies; non-comparative designs; editorials; and studies lacking full-text availability or sufficient raw data.

### 2.3 Data sources and search strategy

We systematically searched PubMed, Web of Science, Scopus, Embase, Cochrane Library, CNKI, Wanfang, and VIP databases, supplemented by manual reference tracking. The search strategy combined MeSH terms and free-text words based on the PICOS framework (Supplementary materials), without language restrictions. Specifically, in line with the specific syntax of each database, MeSH terms and free-text words corresponding to the four core concepts—"cardiopulmonary resuscitation (CPR),” “ethnicity/race,” “comorbidities/past medical history,” and “body mass index (BMI)”—were first connected using the “OR” operator respectively, and then combined with the “AND” operator to retrieve literature relevant to the target factors. Due to space constraints, the detailed search strategies for each database are provided in the Supplementary materials. Two independent researchers performed study selection, with disagreements resolved by a third reviewer.

### 2.4 Data extraction and quality assessment

Data extraction included study characteristics (study, country,period, classification, population, survival to discharge rate, study type and so on), demographics, survival outcomes, and adjusted effect estimates (RR/OR with 95%CIs). Study quality was assessed using the Newcastle-Ottawa Scale (NOS), with high-quality studies defined as NOS ≥ 6. Neurological outcomes were standardized to dichotomous variables (favorable = CPC 1-2/mRS 0–2; Table in supplementary materials).

### 2.5 Statistical analysis

Analyses were performed using RevMan 5.3 and Stata/MP 18.0. We calculated pooled effect sizes (95%CIs) and assessed heterogeneity using the *I*^2^ statistic. Specifically, due to the presence of substantial heterogeneity (*I*^2^ > 50%), we employed random-effects models to account for between-study variability, as recommended by the Cochrane Handbook for Systematic Reviews of Interventions (18). For our outcome measures, which included favorable indicators such as survival rate and good neurological function, and given that the included studies were retrospective in nature, we used Odds Ratio (OR) as the effect measure to quantify the ratio of event occurrence probabilities across different factors. This approach is specifically recommended for meta-analyses of retrospective studies and dichotomous outcomes, as it provides a reliable estimate of relative risk when true incidence rates are unavailable. Sensitivity analyses, meta-regression, and publication bias assessment were also conducted.

## 3 Results

### 3.1 Study selection

#### 3.1.1 Ethnicity

**Figure 1:**
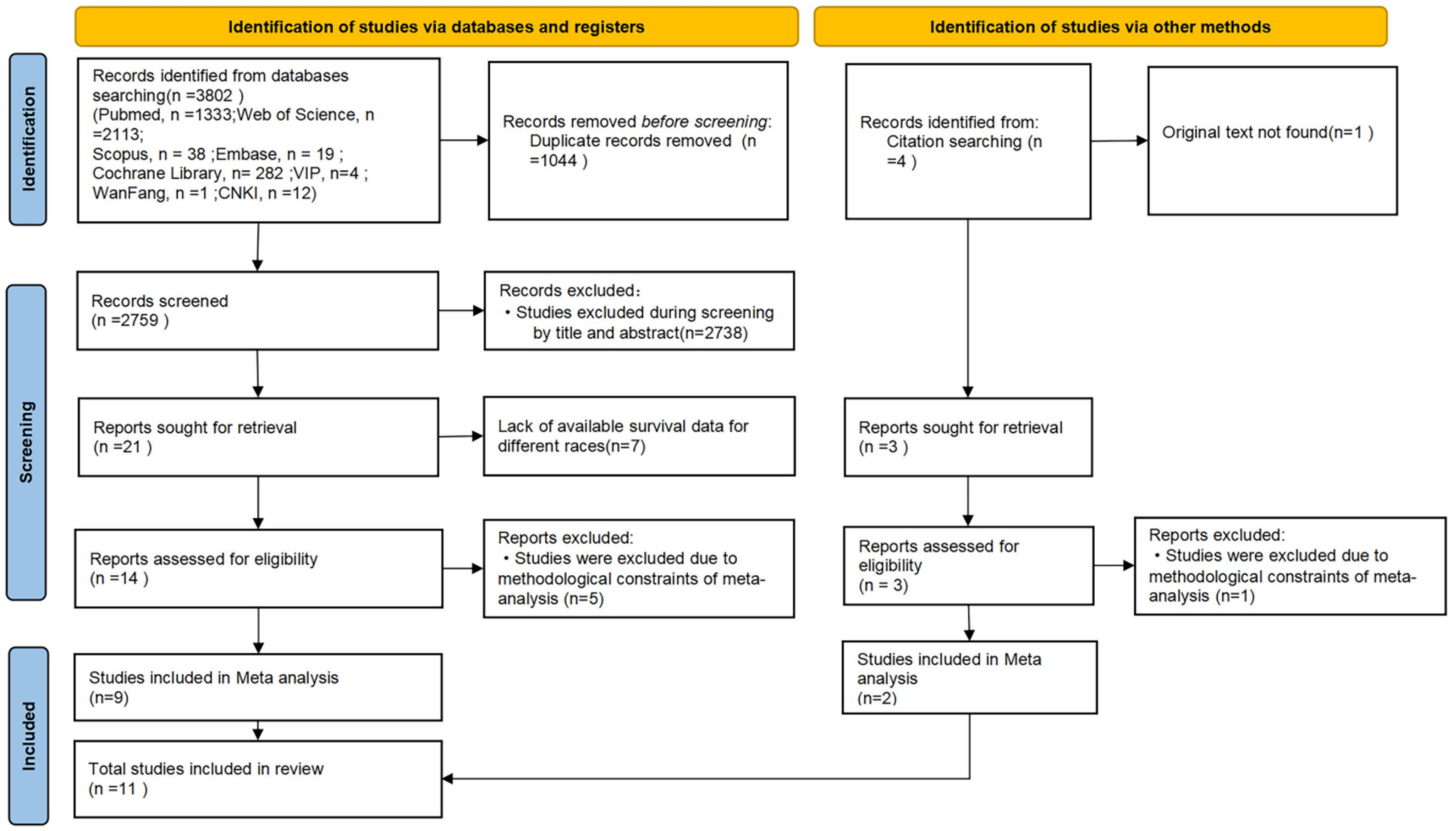
Flowchart of literature screening for ethnicity factors.

Figure 1 delineates the systematic literature selection process of ethnic disparities in CPR outcomes. A total of 3,802 records were identified through database searches, supplemented by 4 additional studies retrieved from the reference lists of included articles, 17 studies (10, 19–34) addressing racial/ethnic disparities in resuscitation outcomes were retained following rigorous eligibility assessment. Notably, 11 of these investigations (10, 19, 20, 22–26, 29, 31, 34) specifically focused on comparative analyses of survival outcomes between Black and White populations following CPR, all conducted in the United States. Detailed study characteristics are presented in Table 1 After comprehensive quality appraisal of observational studies using the Newcastle-Ottawa Scale (NOS), 11 studies comparing post-CPR outcomes between Black and White patient cohorts were ultimately included in the meta-analysis. All included studies achieved a quality score of ≥6 out of 9, a widely accepted threshold in systematic reviews and meta-analyses indicating high methodological quality, which confirms their robustness for subsequent quantitative synthesis.

**Table 1:** Characteristics of studies related to ethnicity factors.

Study	Country	Study period	Ethnicity classification	Population by ethnicity	Survival to discharge rate	Study type
Becker et al. (19)	Chicago, USA	1987.1.1–1988.12.31	Black, White	Total: 6451 (Black: 2910, White: 3207, Other: 334)	Black: 0.8%, White: 2.6%	Cohort Study
Cowie et al. (20)	Seattle, USA	1984.5.30–1986.7.31	Black, White	Total: 977 (Black: 117, White: 860)	Black: 9.4%, White: 17.1%	Cohort Study
Ebell et al. (21)	Monaco	1990.4–1993.6 (Hospital A) 1990.4–1992.6 (Hospitals B & C)	Black, Non-Black	Total: 656 (Black: 398, White: 258)	Black: 3%, Non-Black: 9%	Multicenter Cohort
Chu et al. (22)	Michigan, USA	1991.1.1–1994.12.31	Black, White	Total: 1690 (Black: 223, White: 1467)	Black: 7%, White: 6%	Multicenter Cohort
Sayegh et al. (23)	Michigan, USA	1991–1996	Black, White	Total: 1317 (Black: 378, White: 939)	Black: 2.9%, White: 5.8%	Multicenter Cohort
Galea et al. (24)	New York, USA	2002.4.1–2003.3.31	Hispanic, Black, White, Other	Total: 4653 (Hispan: 636, Black: 1257, White: 1908, Other: 252)	Hispanic: 2%, Black: 1%, White: 3%	Multicenter Cohort
Chan et al. (25)	USA	2000.1.1–2008.2.29	Black, White	Total: 10011 (Black: 1883, White: 8128)	Black: 25.2%, White: 37.4%	Multicenter Cohort
Raina et al. (26)	USA	2000–2007	White, Black	Total: 68115 (White: 60173, Black: 7942)	Black: 30%, White: 33%	Multicenter Cohort
Wilde et al. (27)	Illinois, USA	1996.1.1–2004.12.31	Black, White	Total: 3869 (Black: 353, White: 3516)	(ROSC Rate) Black: 19.8%, White: 26.3%	Cohort Study
Moon et al. (28)	Arizona, USA	2011.1–2012.12.31	Hispanic, Non-Hispanic	Total: 4821 (Hispanic: 215, Non-Hispanic White: 3178, Integrated Community: 3178)	Hispanic: 4.9%, Non-Hispanic: 10.7%, Non-Hispanic White: 10.8%	Cohort Study
Joseph et al. (29)	USA	2000.1.1–2014.12.31	Black, White	Total: 112139 (Black: 30241, White: 81898)	Black: 21.4%, White: 23.2%	Cohort Study
Agerström et al. (30)	Sweden	2005–2018.8.20	Nordic, African, Middle Eastern, Other	Total: 24217 (Nordic: 22266, African: 110, Middle Eastern: 437, Other: 1404)	Nordic: 52.2%, African: 50%, Middle Eastern: 64.3%, Other: 54.5%	Cohort Study
Huebinger et al. (31)	Texas, USA	2014–2020	Black, White, Hispanic/Latino	Total: 8363 (Black: 2850, White: 4825, Hispanic/Latino: 2682)	Black: 36.1%, White: 39.7%, Hispanic/Latino: 32.8%	Cohort Study
Gupta et al. (32)	USA	2013–2021	Asian, White	Total: 278989 (Asian:14835, White:264154)	Asian: 8.2%, White: 10.3%	Cohort Study
Shah et al. (33)	UK	2003.4.1–2007.3.31	White, South Asian	Total: 3161 (White:1995, South Asian:183)	White: 8.7%, South Asian:8.9%	Cohort Study
Rabia et al. (34)	USA	2000–2009	Black, White	Total: 76385 (Black: 19236, White: 57149)	White: 12.7%, Black:10.3%	Cohort Study
Brooks et al. (10)	USA	2004–2010	Black, White	Total: (Black: 2512, White: 11620)	White: 15.8%, Black:11.6%	Cohort Study

ROSC Rate: Return of Spontaneous Circulation Rate.

#### 3.1.2 Past medical history

**Figure 2:**
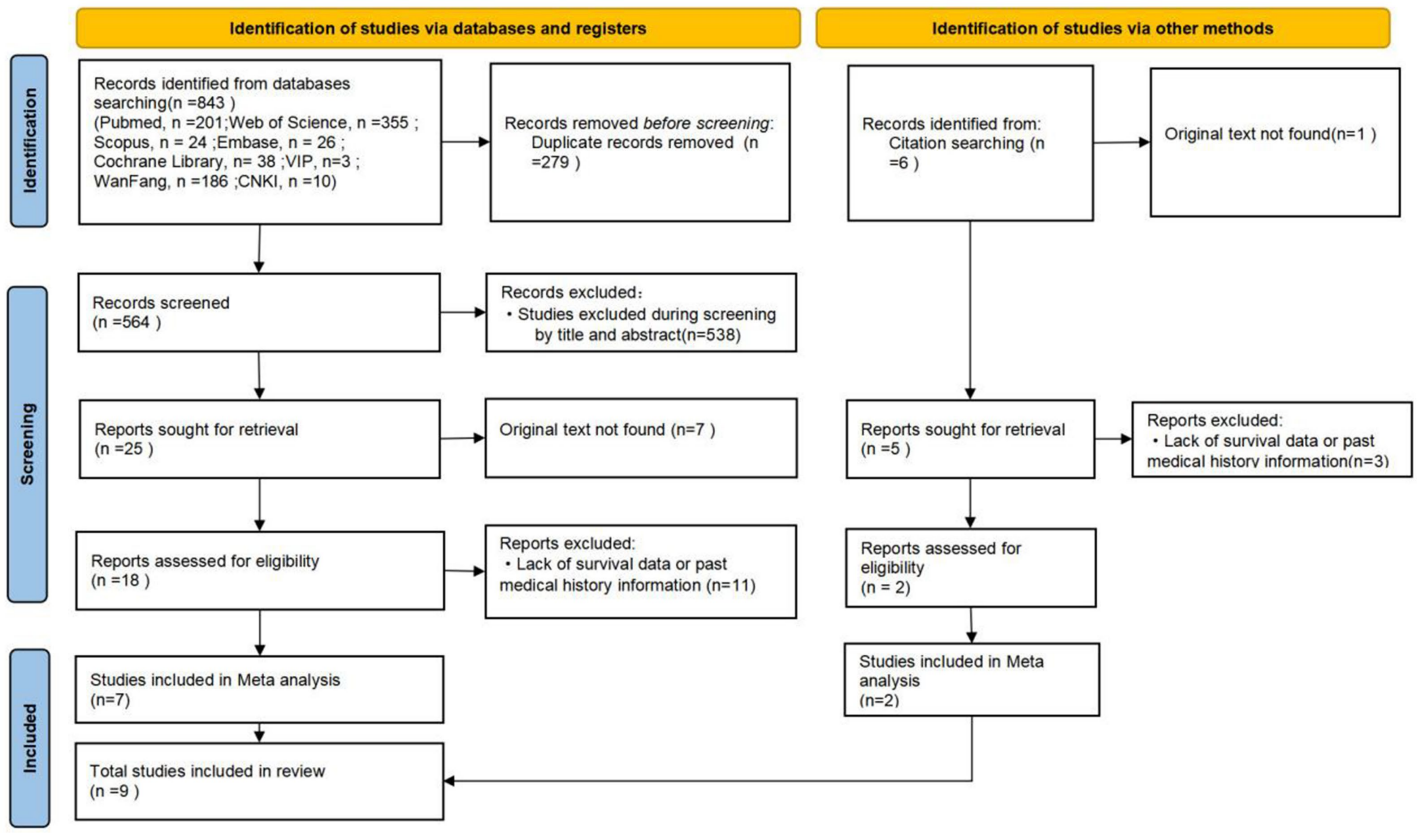
Flowchart of literature screening for medical history factors.

Figure 2 presents a PRISMA-compliant flow diagram for the selection of past medical history-related literature. A total of 843 records were identified through database searches, supplemented by 6 additional studies retrieved from the reference lists of included articles., with nine studies meeting the inclusion criteria (35–43). These observational studies examined the association between pre-existing comorbidities and survival outcomes following CPR. Summarized in Table 2 are the key characteristics of the included studies: Pre-existing Medical Conditions Analysis. Exclusion criteria comprised: (1) non-CPR-related content, (2) incomplete survival data, (3) unspecified comorbidities, and (4) unavailable full-text access.

**Table 2:** Characteristics of studies related to medical history factors.

Study	Country	Study period	Medical history involved in the study	Population by medical history	Survival to discharge rate	Study type
Roth et al. (35)	Israel	1987.7–1998.12	Hypercholesterolemia	With: 115, Without: 883	With: 3.5%, Without: 15.4%	Cohort Study
Zhao et al. (36)	China	2001.4–2006.4	Cardiovascular disease, Cerebrovascular disease, Respiratory system diseases	With: 91, Without: 132	With: 35.2%, Without: 46.7%	Cohort Study
Chen et al. (37)	China	2010.9–2013.9	Cardiovascular diseases, Cerebrovascular disease, Pulmonary diseases, Renal failure, Gastrointestinal bleeding, Chronic cholecystitis with gallstones, acute pancreatitis	With: 73, Without: 13	With: 21.9%, Without: 30.8%	Cohort Study
Bastos et al. (38)	Portugal	2006.10–2018.7	Diabetes Mellitus	With: 15, Without: 45	With: 26.7%, Without: 66.7%	Cohort Study
Nehme et al. (40)	Australia	2007.1.1–2015.6.30	Diabetes mellitus	With: 2438, Without: 9436	With: 6.8%, Without: 13.3%	Cohort Study
Movahedi et al. (41)	Mashhad, Iran	2014.1–2014.2	Diabetes mellitus	With: 21, Without: 59	With: 19%, Without: 44.1%	Cohort Study
Winther-Jensen et al. (39)	Denmark	2007–2011	Cancer	With: 119, Without: 874	With: 31.1%, Without: 41.6%	Cohort Study
Hägglund et al. (42)	Swedish	2010.1.1–2017.12.31	Cancer	With: 2894, Without: 27269	With: 5.1%, Without: 9.4%	Cohort Study
Saeed et al. (43)	USA	2005–2011	Chronic Kidney Disease	With: 71961, Without: 323620	With: 75%, Without: 72%	Cohort Study

#### 3.1.3 Body mass index

**Figure 3:**
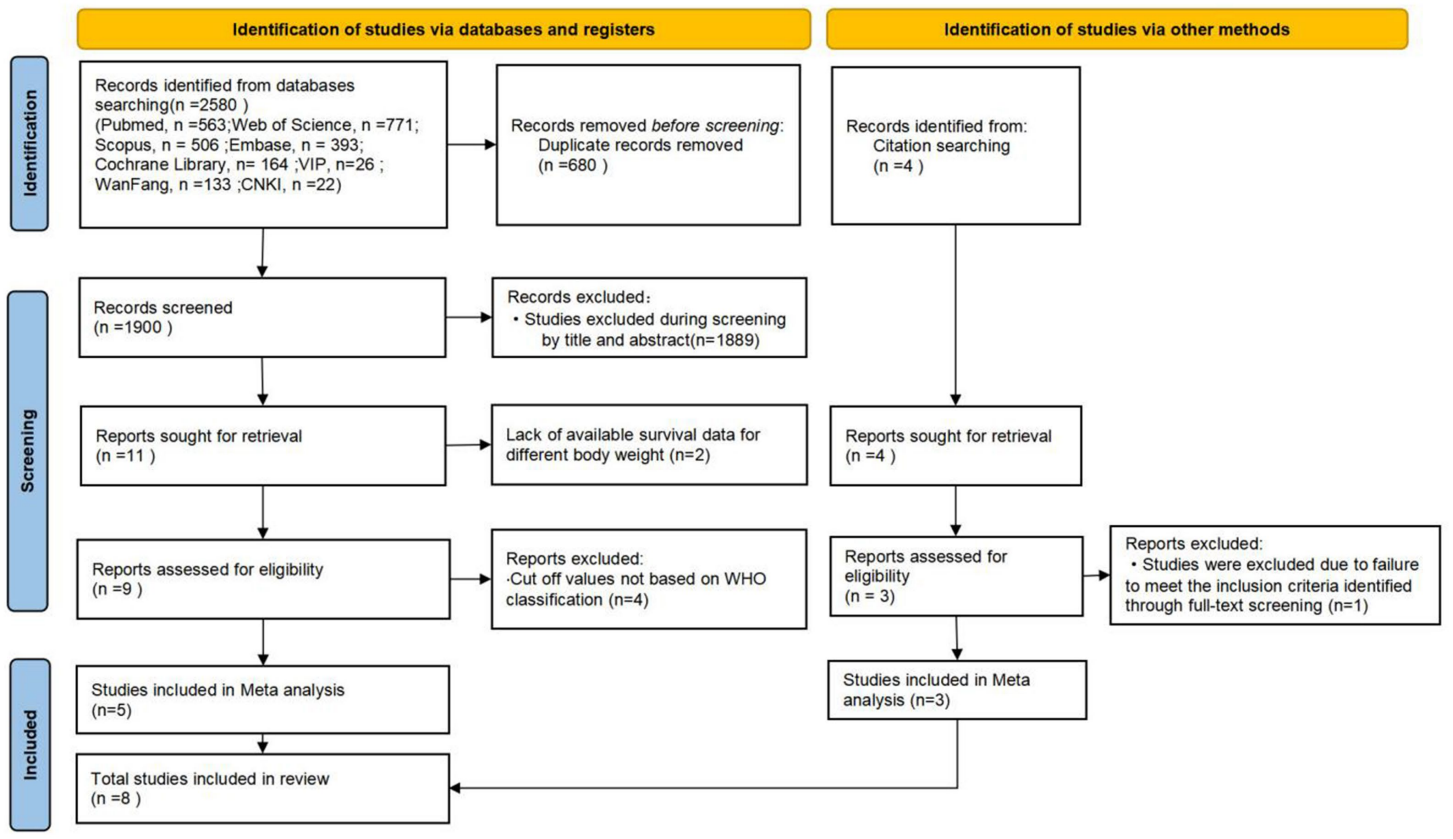
Flowchart of literature screening for BMI factors.

Figure 3 presents a PRISMA-compliant flow diagram for the selection of BMI-related literature. A total of 2,580 records were identified through database searches, supplemented by 4 additional studies retrieved from the reference lists of included articles. After rigorous evaluation, eight studies meeting predefined eligibility criteria were included for quantitative synthesis (44–51), with their key characteristics summarized in Table 3. Exclusion criteria required adherence to WHO BMI classification (18.5–24.9 kg/m^2^ normal range) to ensure standardized data comparability.

**Table 3:** Characteristics of studies related to BMI factors.

Study	Country	Study period	BMI classification	Population by BMI	Survival rate	Good neurological prognosis	Study type
Aoki et al. (44)	Japan	2019–2021	Underweight, Normal, Overweight, Obese	Underweight: 110, Normal: 829, Overweight: 281, Obese: 106	NR	(30 day survival rate) Underweight: 9.1%, Normal: 9.6%, Overweight: 10.3%, Obese: 1.9%	Cohort Study
Lee et al. (45)	South Korea	2015.10–2018.6	Underweight, Normal, Overweight, Obese	Underweight: 75, Normal: 333, Overweight: 163, Obese: 34	(Discharge survival rate) Underweight: 37.3%, Normal: 52.9%, Overweight: 49.7%, Obese: 52.9%	(Discharge neurological prognosis) Underweight: 14.7%, Normal: 36.0%, Overweight: 34.4%, Obese: 44.1%	Cohort Study
Wang et al. (46)	Taiwan	2006–2015	Underweight, Normal, Overweight, Obese	Underweight: 85, Normal: 268, Overweight: 204, Obese: 91	NR	(Discharge neurological prognosis) Underweight:10.6%, Normal: 10.1%, Overweight: 3.9%, Obese: 3.3%	Cohort Study
Aoki et al. (47)	Japan	2012.1–2012.12	Underweight, Normal, Overweight, Obese	Underweight: 69, Normal: 312, Overweight: 176, Obese: 80	(30 day survival rate) Underweight: 40.6%, Normal: 42.0%, Overweight: 38.1%, Obese: 26.2%	(30 day neurological prognosis) Underweight: 23.2%, Normal: 29.2%, Overweight: 20.5%, Obese: 16.2%	Cohort Study
Testori et al. (48)	Vienna	1992.1–2007.12	Underweight, Normal, Overweight, Obese	Underweight: 37, Normal: 768, Overweight: 781, Obese: 329	month survival rate) Underweight: 45.9%, Normal: 46.9%, Overweight: 52.5%, Obese: 50.8%	(6-month neurological prognosis) Underweight: 40.5%, Normal: 47.9%, Overweight: 55.4%, Obese: 51.1%	Cohort Study
Jain et al. (49)	USA	2006.1.1–2007.12.31	Underweight, Normal, Overweight, Obese	Underweight:1437, Normal: 6935, Overweight: 5919, Obese: 6946	Underweight: 12.9%, Normal: 18.3%, Overweight: 22.0%, Obese: 55.0%	NR	Cohort Study
Bunch et al. (50)	Rochester, USA	1990.11–2006.9	Normal, Overweight, Obese	Normal: 68, Overweight: 78, Obese: 67	(Discharge survival rate) Normal: 45.6%, Overweight: 46.2%, Obese: 47.8%	(Discharge neurological prognosis) Normal: 45.6%, Overweight: 46.2%, Obese: 47.8%	Cohort Study
Ikemura et al. (51)	USA	2006–2012	Underweight, Normal, Overweight, Obese	Underweight: 3617, Normal: 17713, Overweight: 15523, Obese: 9383	Underweight: 24.4%, Normal: 36.6%, Overweight: 41.7%, Obese: 41.9%	Underweight: 7.2%, Normal: 9.7%, Overweight: 10.7%, Obese: 12.1%	Cohort Study

NR, No Report.

### 3.2 Meta-analysis finding

#### 3.2.1 Ethnicity

**Figure 4:**
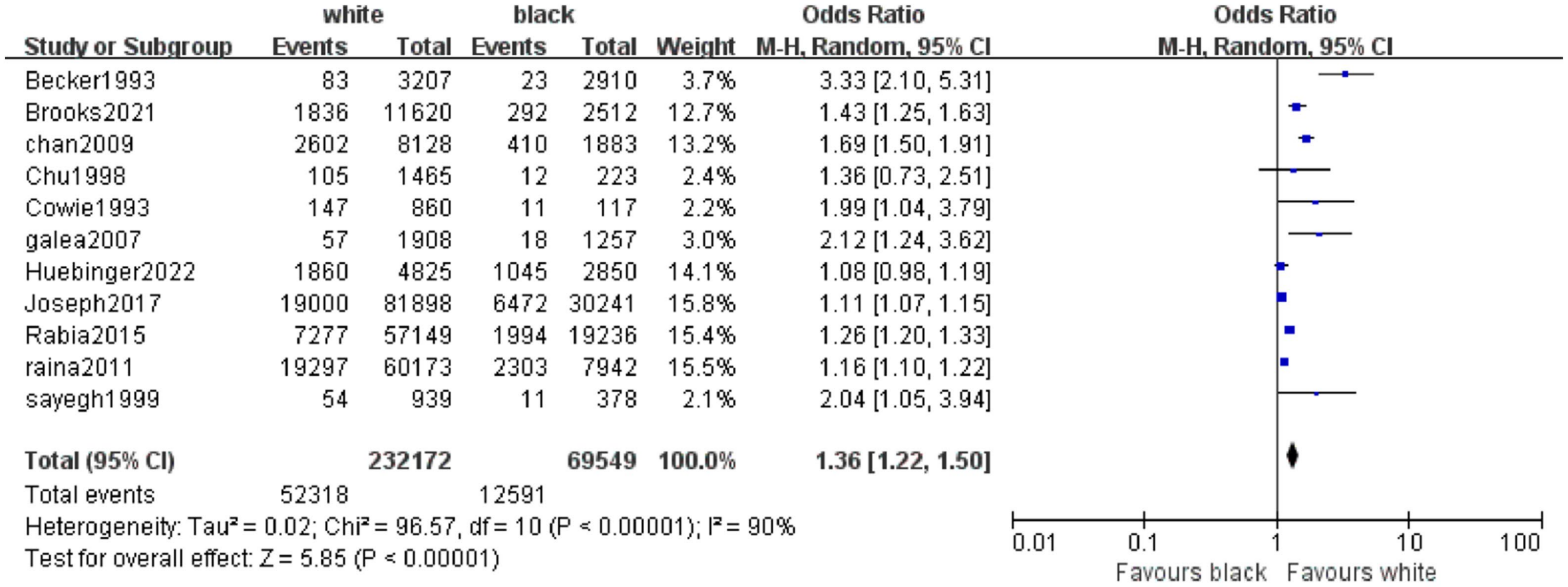
Forest plot of meta-analysis on hospital discharge survival rate after cardiopulmonary resuscitation between White and Black Racial Groups. In this forest plot, “Study or Subgroup” lists the included studies. The “white” and “black” columns present the number of events (i.e., survivors) and the total number of patients in White and Black cohorts, respectively. The “Weight” column indicates the weight of each study in the pooled analysis (a larger weight means a stronger influence on the combined result). The “Odds Ratio (M-H, Random, 95% CI)” column shows the odds ratio (OR) and 95% confidence interval (CI) for each individual study and the overall synthesis. Graphically, the size of each square corresponds to the study’s weight, the square’s position represents the study’s OR, and the length of the horizontal line reflects the 95% CI. The diamond at the bottom denotes the overall pooled effect size, with its position and horizontal span corresponding to the overall OR and 95% CI. Results indicate that White patients had a significantly higher hospital discharge survival rate after cardiopulmonary resuscitation than Black patients, with a pooled OR of 1.36 (95% CI: 1.22–1.50, *p* < 0.00001). Significant heterogeneity was observed among studies (*I*^2^ = 90%, *p* < 0.00001), so a random-effects model was applied for analysis.

**Figure 5:**
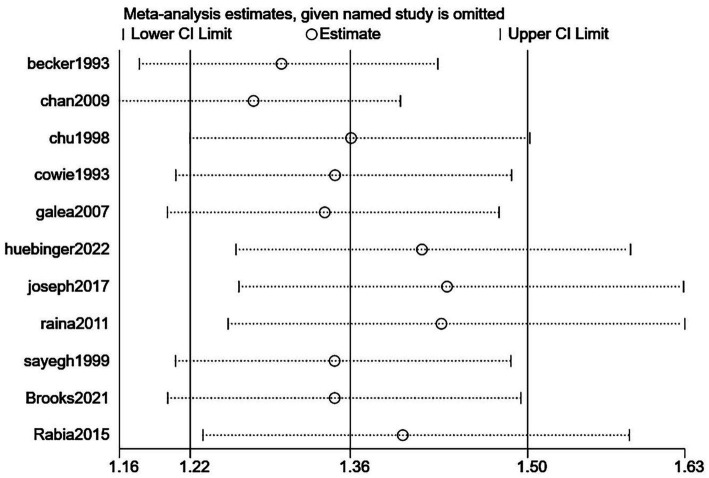
Sensitivity analysis plot for the impact of racial factors on hospital discharge survival rate after cardiopulmonary resuscitation. This plot is a sensitivity analysis for the impact of racial factors (White vs. Black) on hospital discharge survival rate after cardiopulmonary resuscitation. The horizontal axis represents the range of pooled odds ratios (ORs), and the vertical axis lists the studies that were sequentially excluded. “Lower CI Limit” and “Upper CI Limit” denote the lower and upper bounds of the 95% confidence interval of the pooled effect after excluding the corresponding study, respectively, and “Estimate” is the pooled effect estimate after excluding that study. The results show that after excluding each study one by one, the 95% confidence interval of the pooled effect remains within 1.16–1.63, and all estimates fluctuate around the overall pooled OR (1.36), indicating that the findings of this study regarding the impact of race on hospital discharge survival rate after cardiopulmonary resuscitation are robust.

**Figure 6:**
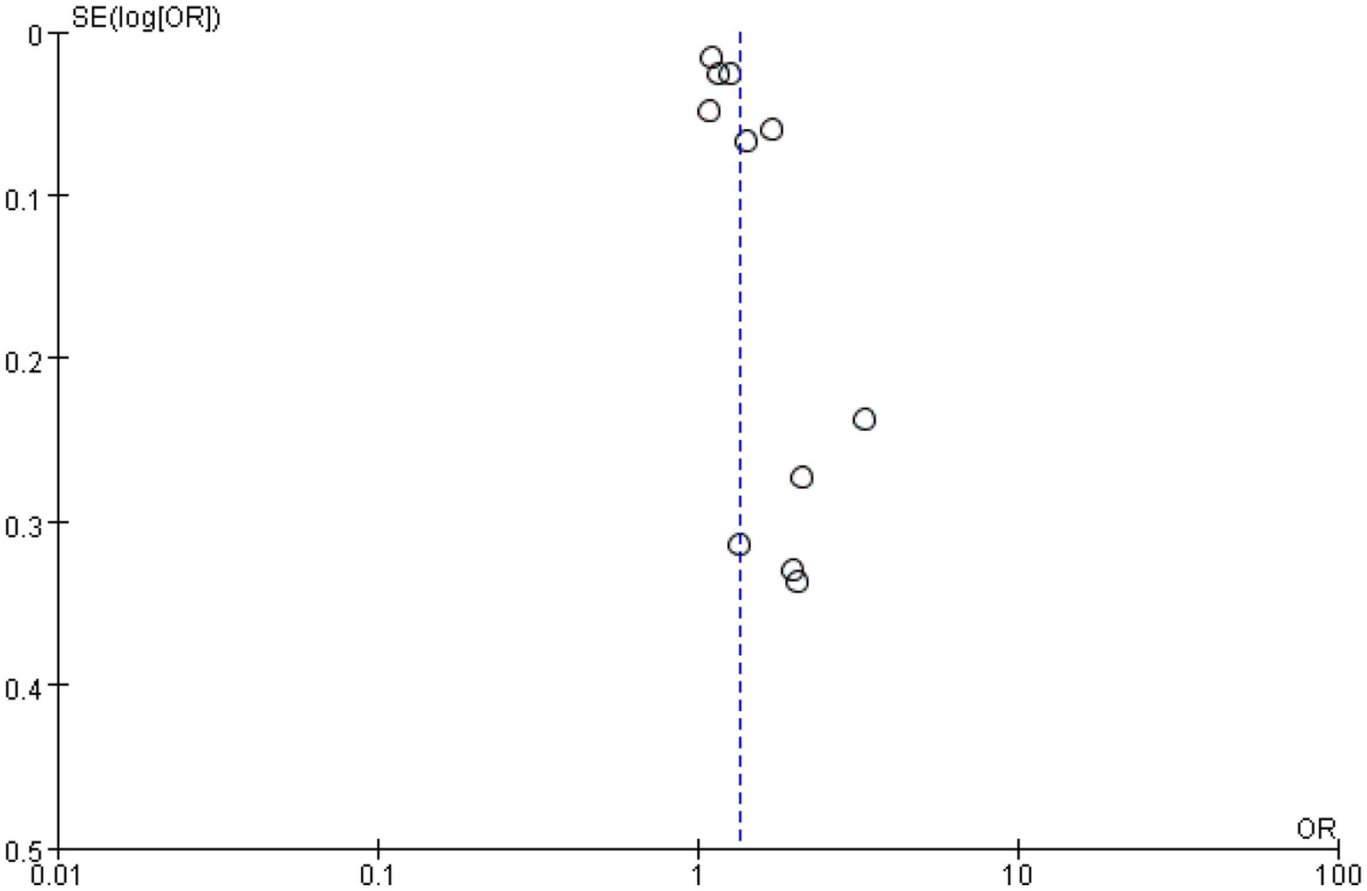
Funnel plot for assessing publication bias of racial factors on hospital discharge survival rate after cardiopulmonary resuscitation. This funnel plot assesses publication bias in the analysis of the impact of racial factors (White vs. Black) on hospital discharge survival rate after cardiopulmonary resuscitation. The horizontal axis represents the odds ratio (OR), and the vertical axis represents the standard error of the logarithmic odds ratio [SE(log[OR])]. Each point represents an included study, and its position reflects the relationship between the study’s effect size (OR) and precision (standard error)—larger sample sizes correspond to smaller standard errors and higher-positioned points, while smaller sample sizes correspond to larger standard errors and lower-positioned points. The blue dashed line corresponds to the pooled effect size of racial factors in this study (OR = 1.36). Although the distribution of study points is somewhat scattered, there is no obvious unilateral absence, indicating a low risk of publication bias.

The primary outcome was hospital discharge survival rate. White patients had a significantly higher post-CPR hospital discharge survival rate than Black patients, with a pooled odds ratio (OR) of 1.36 (95% confidence interval [CI]: 1.22–1.50; *p* < 0.00001) (Figure 4). Given significant heterogeneity among different racial groups (*I*^2^ = 90%), the use of a random-effects model was justified. The sensitivity analysis in Figure 5 demonstrated stable results, with all recalculated ORs remaining >1 and 95% CIs consistently showing statistical significance, confirming the robustness of the findings. Figure 6 was used to assess publication bias. This funnel plot has the odds ratio (OR) on the horizontal axis and the standard error of the logarithmic odds ratio [SE (logOR)] on the vertical axis. Each point represents an included study, and its position reflects the relationship between the study’s effect size (OR) and precision (standard error)—larger sample sizes correspond to smaller standard errors and higher-positioned circles, while smaller sample sizes correspond to larger standard errors and lower-positioned points. The blue dashed line corresponds to the pooled effect size for the racial factor in this study (OR = 1.36). Ideally, study points should be roughly symmetrically distributed around this dashed line, forming a funnel shape. Although the distribution of study points here is somewhat scattered, there is no obvious unilateral absence, indicating a low risk of publication bias.

**Figure 7:**
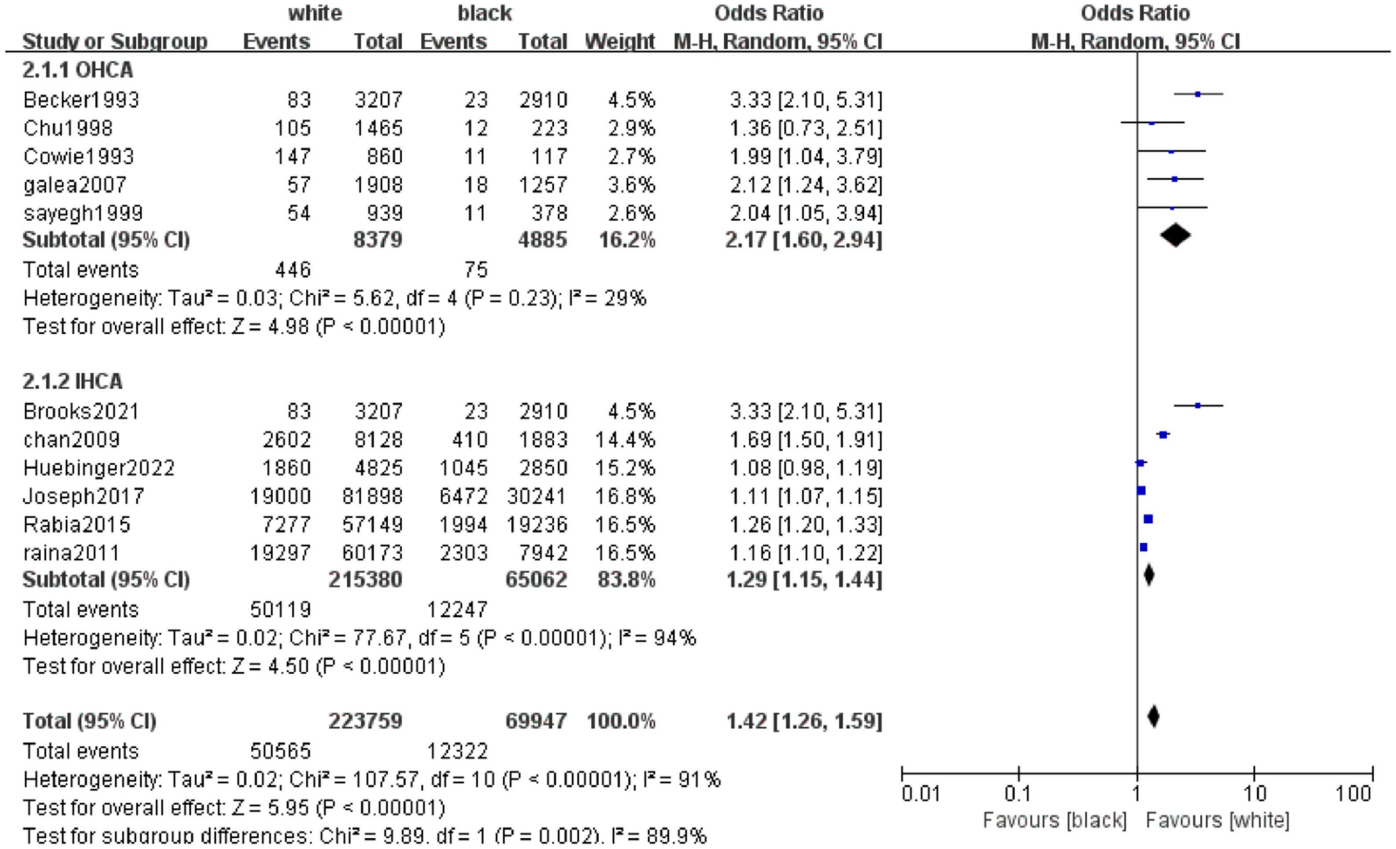
Forest plot of meta-analysis on the impact of ethnicity (Black vs. White) on survival outcomes after cardiopulmonary resuscitation in OHCA and IHCA subgroups. This forest plot illustrates the meta-analysis results comparing survival outcomes after CPR between Black and White patients, stratified by two cardiac arrest scenarios: OHCA and IHCA. The column “Study or Subgroup” denotes the included studies or subgroup categories. Under the “white” column, “Events” and “Total” represent the number of survival events and the total number of participants in the White group, respectively; similarly, “Events” and “Total” under the “black” column refer to those in the Black group. The “Weight” column indicates the weight of each study in the meta-analysis. The “Odds Ratio” column employs the Mantel–Haenszel (M-H) random-effects model to calculate and present the odds ratio (OR) and its 95% confidence interval (95% CI), which is used to compare survival differences between Black and White populations (an OR > 1 suggests a higher survival rate in the White group, while an OR < 1 suggests a higher survival rate in the Black group). In the forest plot, the size of each square is proportional to the study’s weight, the horizontal line represents the 95% CI, and the diamond denotes the pooled OR and 95% CI for the subgroup or the overall analysis. For heterogeneity assessment, the “Heterogeneity” row includes Tau^2^ (heterogeneity variance), Chi^2^ (chi-square test statistic), df (degrees of freedom), and *I*^2^ (percentage of heterogeneity; *I*^2^ < 50% indicates low heterogeneity, whereas *I*^2^ ≥ 50% indicates moderate to high heterogeneity). The “Test for overall effect” row reports the *Z* value and *p* value to determine the statistical significance of the pooled effect. The axis scales “0.01,” “0.1,” “1,” “10,” and “100” correspond to OR values, with “Favours [black]” indicating that OR values in this range support better survival in the Black group and “Favours [white]” indicating better survival in the White group. The results show that in the OHCA subgroup, White patients had a significantly higher survival rate after CPR than Black patients (OR = 2.17, 95% CI [1.60, 2.94], *p* < 0.00001), with low heterogeneity (*I*^2^ = 29%). In the IHCA subgroup, White patients also had a significantly higher survival rate (OR = 1.29, 95% CI [1.15, 1.44], *p* < 0.00001), but with high heterogeneity (*I*^2^ = 94%), indicating substantial differences among studies in this subgroup, and the conclusion should be interpreted cautiously.

OHCA and IHCA are important situational factors influencing the impact of ethnicity on patient survival outcomes, we conducted subgroup analyses for the included studies of Black and White patients stratified by OHCA and IHCA. As shown in Figure 7, the results showed that in the OHCA scenario, White patients had a significantly higher survival rate than Black patients, with an odds ratio (OR) = 2.17, 95% confidence interval (CI) [1.60, 2.94], *p* < 0.00001; in the IHCA scenario, White patients also had a significantly higher survival rate than Black patients, OR = 1.29, 95% CI [1.15, 1.44], *p* < 0.00001. Among them, the heterogeneity I ^2^ of the OHCA subgroup is 29%, indicating good homogeneity and reliable results; however, the IHCA subgroup still showed higher *I*^2^, which may be due to the presence of multiple confounding factors in in-hospital cardiac arrest scenarios. However, the data recorded by the included studies lacked consistency and clarity, resulting in no further analysis.

#### 3.2.2 Past medical history

**Figure 8:**
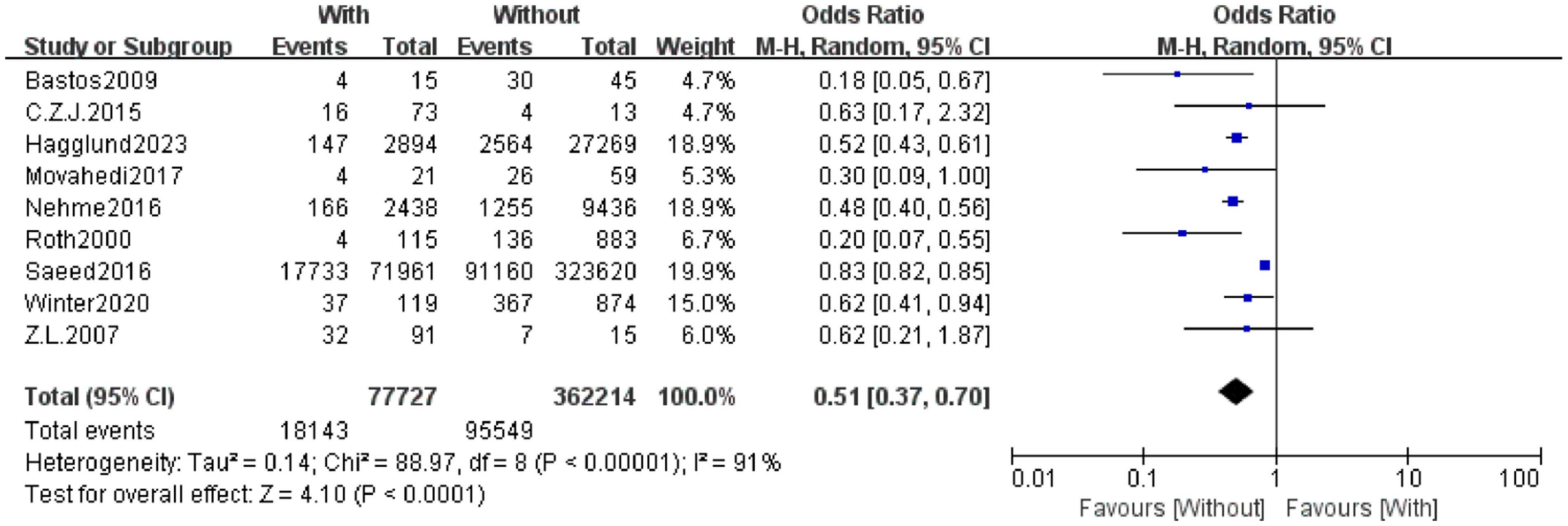
Forest plot of hospital discharge survival rate after cardiopulmonary resuscitation between patients with and without past medical history. This forest plot presents the meta-analysis results of the impact of past medical history on hospital discharge survival rate after cardiopulmonary resuscitation. “Study or Subgroup” lists the included studies; the “With” column shows the number of events (survivors) and total patients in the group with past medical history, while the “Without” column shows those in the group without past medical history; “Weight” indicates the weight of each study in the pooled analysis (a larger weight means a stronger influence on the combined result); “Odds Ratio (M-H, Random, 95% CI)” presents the odds ratio (OR) and 95% confidence interval (CI) for each individual study and the overall synthesis. Graphically, the size of each square corresponds to the study’s weight, the square’s position represents the study’s OR, and the length of the horizontal line reflects the 95% CI. The diamond at the bottom denotes the overall pooled effect size, with its position and horizontal span corresponding to the overall OR and 95% CI. Results indicate that patients without past medical history had a significantly higher hospital discharge survival rate after cardiopulmonary resuscitation than those with a history (pooled OR = 0.51, 95% CI: 0.37–0.70, *p* < 0.0001). Significant heterogeneity was observed among studies (*I*^2^ = 91%, *p* < 0.00001), so a random-effects model was applied for analysis.

**Figure 9:**
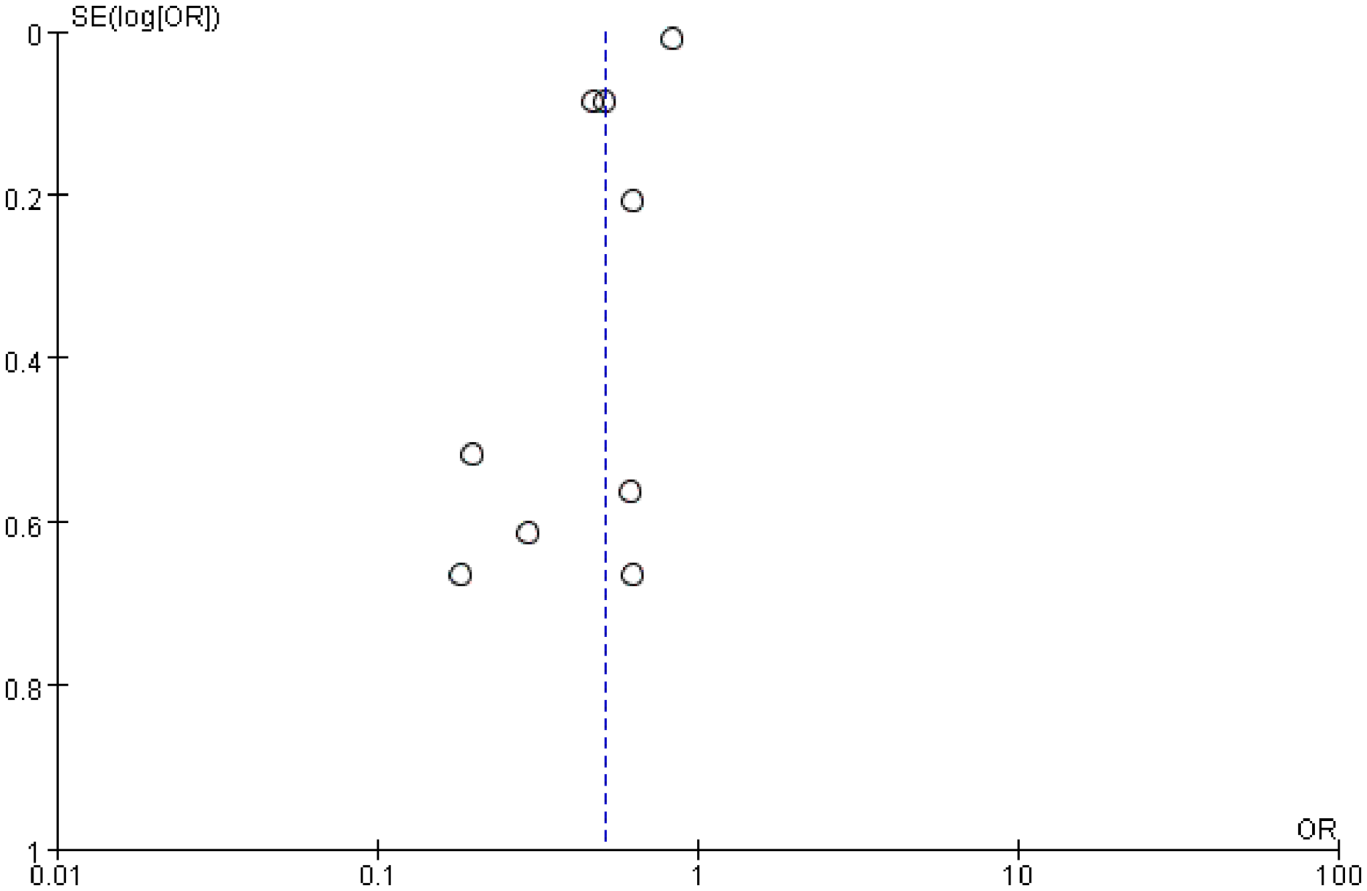
Funnel plot for assessing publication bias of past medical history on hospital discharge survival rate after cardiopulmonary resuscitation. This funnel plot assesses publication bias in the analysis of the impact of past medical history on hospital discharge survival rate after cardiopulmonary resuscitation. The horizontal axis represents the odds ratio (OR), and the vertical axis represents the standard error of the logarithmic odds ratio [SE(log[OR])]. Each point represents an included study, and its position reflects the relationship between the study’s effect size (OR) and precision (standard error)—larger sample sizes correspond to smaller standard errors and higher-positioned points, while smaller sample sizes correspond to larger standard errors and lower-positioned points. The blue dashed line corresponds to the pooled effect size of the past medical history factor in this study (OR = 0.51). The study points show a roughly symmetric distribution trend without obvious unilateral absence, indicating a low risk of publication bias.

**Figure 10:**
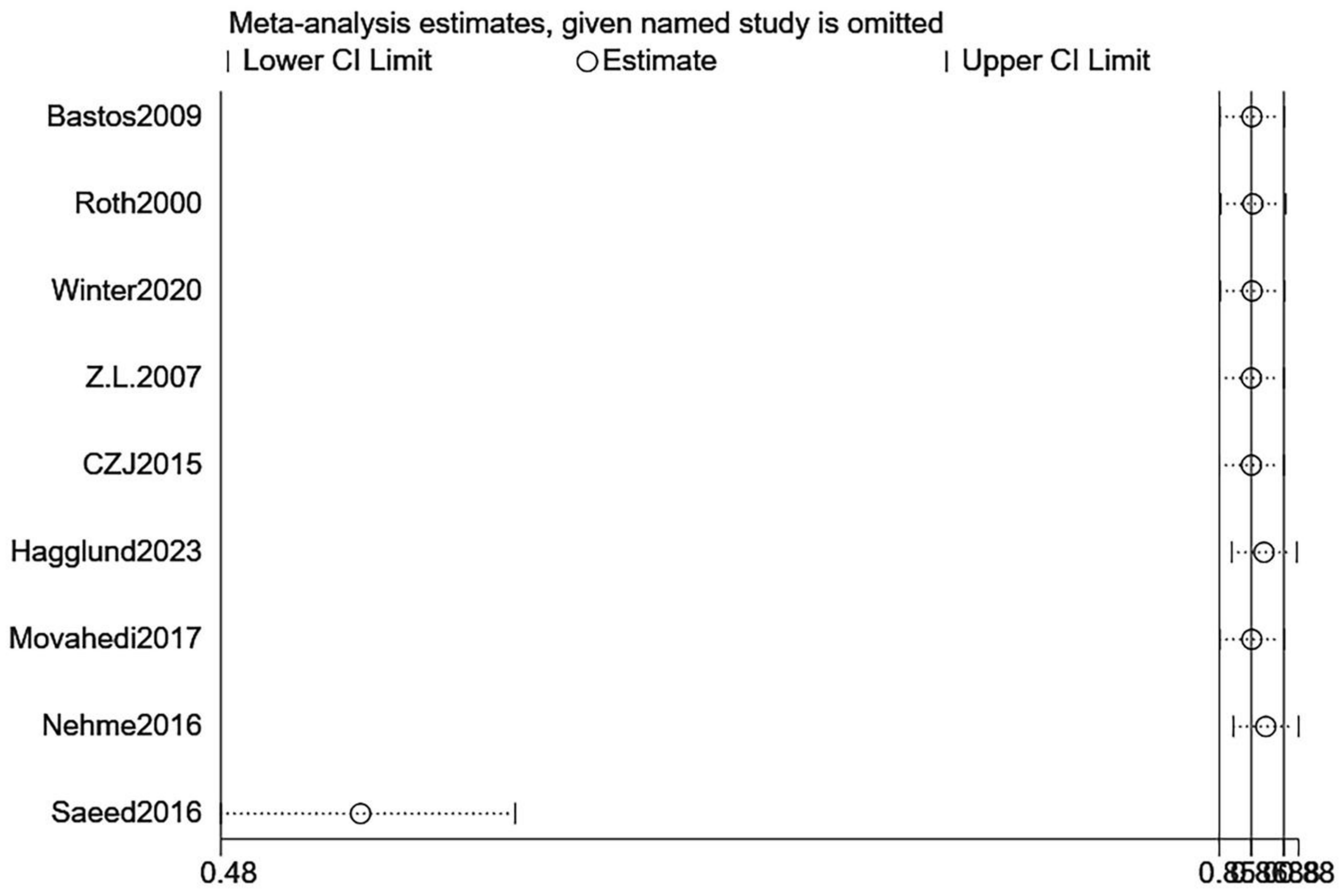
Sensitivity analysis plot for the impact of past medical history on hospital discharge survival rate after cardiopulmonary resuscitation. This plot is a sensitivity analysis for the impact of past medical history on hospital discharge survival rate after cardiopulmonary resuscitation. The vertical axis lists the studies that were sequentially excluded, and the horizontal axis represents the range of pooled odds ratios (ORs). “Lower CI Limit” and “Upper CI Limit” denote the lower and upper bounds of the 95% confidence interval of the pooled effect after excluding the corresponding study, respectively, and “Estimate” is the pooled effect estimate after excluding that study. The results show that after excluding each study one by one, the 95% confidence interval of the pooled effect remains within 0.48–0.88, and all estimates fluctuate around the overall pooled OR (0.51), indicating that the findings of this study regarding the impact of past medical history on hospital discharge survival rate after cardiopulmonary resuscitation are robust.

The primary outcome was survival to hospital discharge. Patients without a recorded past medical history had a significantly higher post-CPR survival rate than those with a history (pooled OR = 0.51, 95% CI: 0.37–0.70; *p* < 0.0001) (Figure 8). Assessment via the funnel plot (Figure 9) revealed a symmetric inverted funnel distribution, indicating a low risk of publication bias; larger studies clustered near the top and center, while smaller studies were distributed at the bottom with greater dispersion. The sensitivity analysis plot (Figure 10) showed that after sequentially excluding each individual study, the pooled effect size and its 95% CI remained < 1, confirming the robustness of the findings.

**Figure 11:**
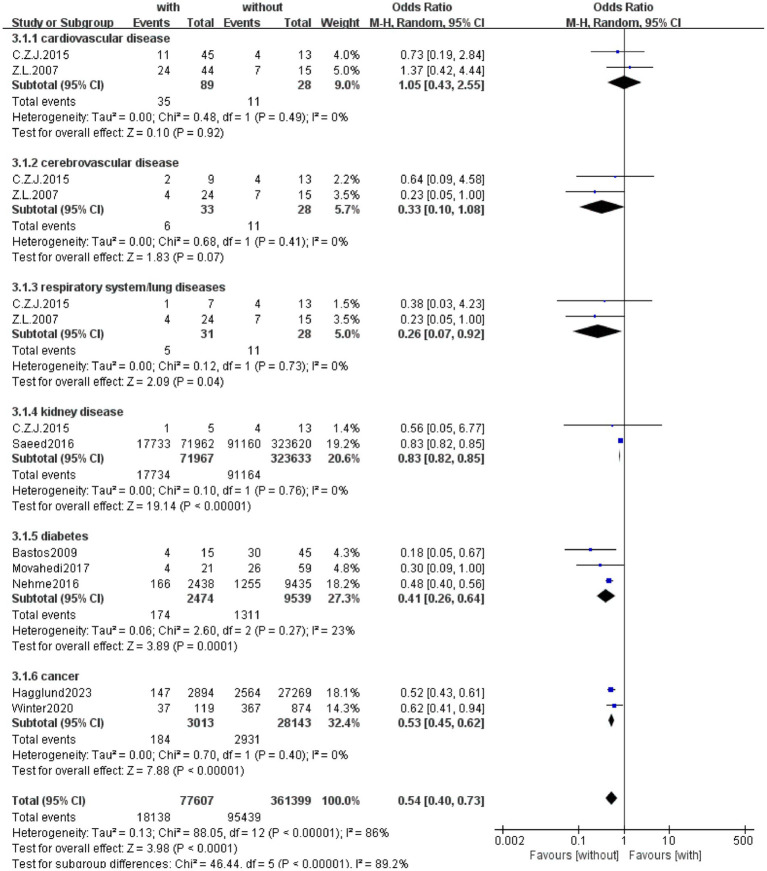
Forest plot of meta-analysis: impact of different pre-existing disease types on survival outcomes after cardiopulmonary resuscitation. This forest plot presents the meta-analysis results on the impact of different pre-existing disease types (cardiovascular disease, cerebrovascular disease, respiratory system/lung diseases, kidney disease, diabetes, cancer) on survival outcomes after CPR. The column “Study or Subgroup” indicates the included studies or disease subgroup categories. Under the “with” column, “Events” and “Total” represent the number of survival events and the total number of participants in the group with the disease, respectively; similarly, “Events” and “Total” under the “without” column refer to those in the group without the disease. The “Weight” column shows the weight of each study in the meta-analysis. The “Odds Ratio” column uses the Mantel–Haenszel (M-H) random-effects model to calculate and present the OR and its 95% CI, which is used to compare survival differences between populations with and without the disease (an OR < 1 suggests a higher survival rate in the group without the disease, while an OR > 1 suggests a higher survival rate in the group with the disease). In the forest plot, the size of each square is proportional to the study’s weight, the horizontal line represents the 95% CI, and the diamond denotes the pooled OR and 95% CI for the subgroup or the overall analysis. The axis scales “0.002,” “0.1,” “1,” “10,” and “500” correspond to OR values, with “Favours [without]” indicating that OR values in this range support better survival in the group without the disease and “Favours [with]” indicating better survival in the group with the disease. The specific subgroup results are as follows: Cardiovascular disease subgroup: Pooled OR = 1.05 (95% CI [0.43, 2.55]), *p* = 0.92, heterogeneity *I*^2^ = 0%; Cerebrovascular disease subgroup: Pooled OR = 0.33 (95% CI [0.10, 1.08]), *p* = 0.07, heterogeneity *I*^2^ = 0%; Respiratory system/lung diseases subgroup: Pooled OR = 0.26 (95% CI [0.07, 0.92]), *p* = 0.04, heterogeneity *I*^2^ = 0%; Kidney disease subgroup: Pooled OR = 0.83 (95% CI [0.82, 0.85]), p < 0.00001, heterogeneity *I*^2^ = 0%; Diabetes subgroup: Pooled OR = 0.41 (95% CI [0.26, 0.64]), *p* = 0.0001, heterogeneity *I*^2^ = 23%; Cancer subgroup: Pooled OR = 0.53 (95% CI [0.45, 0.62]), p < 0.00001, heterogeneity *I*^2^ = 0%; Overall analysis: Pooled OR = 0.54 (95% CI [0.40, 0.73]), *p* < 0.00001.

In order to further explore the impact of specific pre-existing disease types on the survival results of CPR, we conducted a subgroup analysis based on the available data of the included studies, stratified by cardiovascular disease, cerebrovascular disease, respiratory/pulmonary disease, kidney disease, diabetes and cancer. As shown in Figure 11. The overall results indicate that the presence of a specific medical history has an impact on the survival rate of patients, and the insignificant results of cardiovascular and cerebrovascular diseases should be related to the small number of included literature and patients.

#### 3.2.3 Body mass index

**Figure 12:**
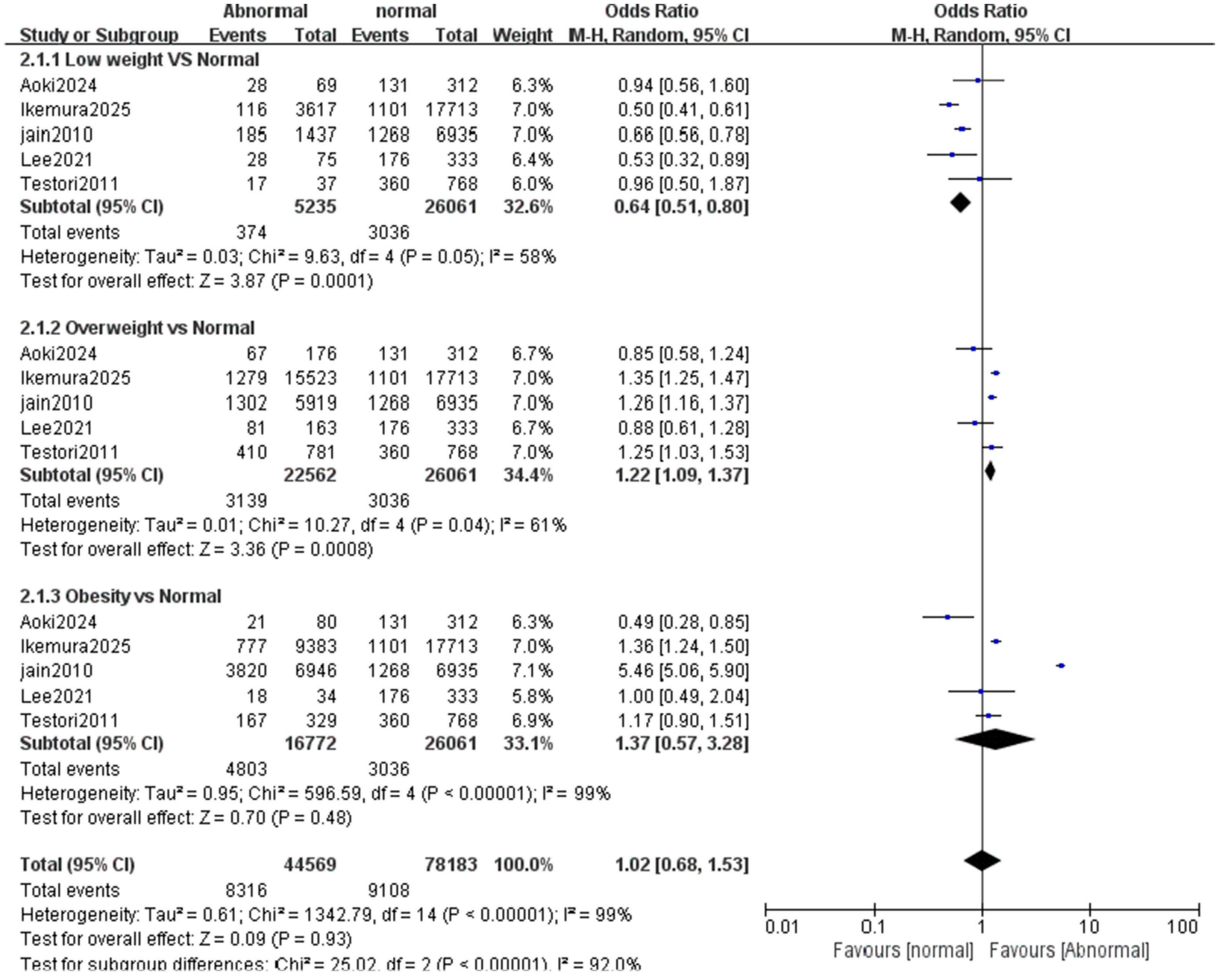
Forest plot of meta-analysis on hospital discharge survival rate after cardiopulmonary resuscitation between different BMI groups (underweight, overweight, obese) and normal weight controls. This forest plot presents the meta-analysis results of hospital discharge survival rate after cardiopulmonary resuscitation among three BMI groups (underweight, overweight, obese) compared with normal weight controls, including 2.1.1 Low weight vs. Normal, 2.1.2 Overweight vs. Normal, and 2.1.3 Obesity vs. Normal subgroups. “Study or Subgroup” lists the included studies; the “Abnormal” column shows the number of events (survivors) and total patients in the underweight, overweight, or obese group, while the “normal” column shows those in the normal weight group; “Weight” indicates the weight of each study in the pooled analysis of the corresponding subgroup (a larger weight means a stronger influence on the subgroup result); “Odds Ratio (M-H, Random, 95% CI)” presents the odds ratio (OR) and 95% confidence interval (CI) for each individual study, each subgroup, and the overall synthesis. Graphically, the size of each square corresponds to the study’s weight in the subgroup, the square’s position represents the study’s OR, and the length of the horizontal line reflects the 95% CI. The diamond at the bottom of each subgroup or the overall section denotes the pooled effect size, with its position and horizontal span corresponding to the pooled OR and 95% CI. Results indicate that compared with the normal weight control group, the survival rate was statistically significantly lower in underweight patients (OR = 0.64, 95% CI 0.51–0.80; p = 0.0001), significantly higher in overweight patients (OR = 1.22, 95% CI 1.09–1.37; *p* = 0.0008), while no significant survival difference was observed between the obese group and the normal weight group (OR = 1.37, 95% CI 0.57–3.28; *p* = 0.48). There was heterogeneity among studies to varying degrees, with *I*^2^ = 58% in the underweight subgroup, *I*^2^ = 61% in the overweight subgroup, *I*^2^ = 99% in the obese subgroup, and *I*^2^ = 99% overall, so a random-effects model was applied for analysis.

**Figure 13:**
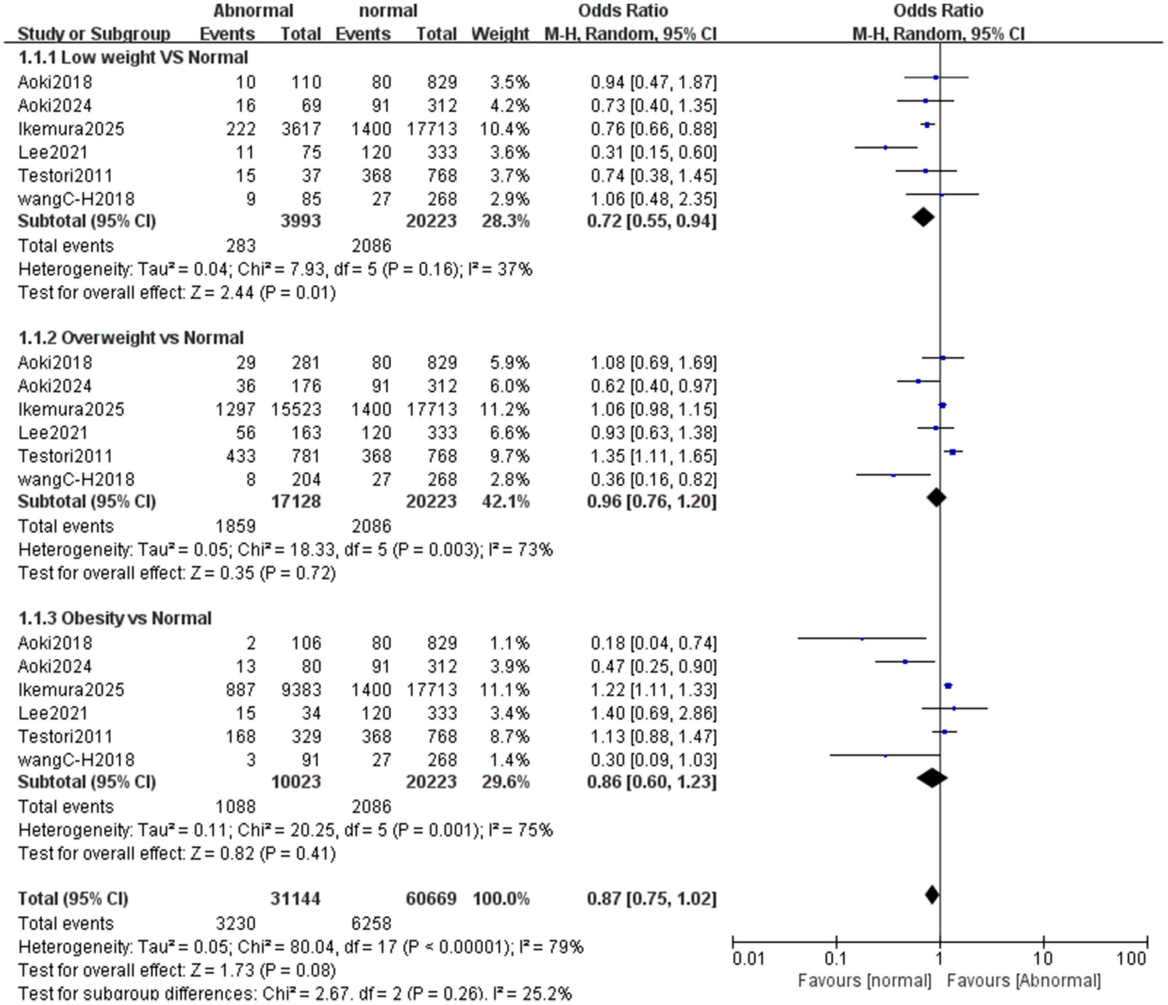
Forest plot of meta-analysis on neurological function scores after cardiopulmonary resuscitation between different BMI groups (underweight, overweight, obese) and normal weight controls. This forest plot presents the meta-analysis results of neurological function scores after cardiopulmonary resuscitation among three BMI groups (underweight, overweight, obese) compared with normal weight controls, including 1.1.1 Low weight vs. Normal, 1.1.2 Overweight vs. Normal, and 1.1.3 Obesity vs. Normal subgroups. “Study or Subgroup” lists the included studies; the “Abnormal” column shows the number of events (patients with satisfactory neurological function scores) and total patients in the underweight, overweight, or obese group, while the “normal” column shows those in the normal weight group; “Weight” indicates the weight of each study in the pooled analysis of the corresponding subgroup (a larger weight means a stronger influence on the subgroup result); “Odds Ratio (M-H, Random, 95% CI)” presents the odds ratio (OR) and 95% confidence interval (CI) for each individual study, each subgroup, and the overall synthesis. Graphically, the size of each square corresponds to the study’s weight in the subgroup, the square’s position represents the study’s OR, and the length of the horizontal line reflects the 95% CI. The diamond at the bottom of each subgroup or the overall section denotes the pooled effect size, with its position and horizontal span corresponding to the pooled OR and 95% CI. Results indicate that compared with the normal weight control group, patients with low weight had significantly poorer neurological function scores (OR = 0.72, 95% CI 0.55–0.94; *p* = 0.01), while no significant differences were observed between the overweight group and the normal weight group (OR = 0.96, 95% CI 0.76–1.20; *p* = 0.72) or between the obese group and the normal weight group (OR = 0.86, 95% CI 0.60–1.23; *p* = 0.41). There was heterogeneity among studies to varying degrees, with *I*^2^ = 37% in the underweight subgroup, *I*^2^ = 73% in the overweight subgroup, *I*^2^ = 75% in the obese subgroup, and *I*^2^ = 79% overall, so a random-effects model was applied for analysis.

The primary outcome was hospital discharge survival rate, and neurological outcome was the secondary outcome. Forest plot analysis showed that, compared with the normal weight control group, the survival rate was statistically significantly lower in underweight patients (OR = 0.64, 95% CI 0.51–0.80; *p* = 0.0001). The survival rate was significantly higher in overweight patients than in normal-weight patients (OR = 1.22, 95% CI 1.09–1.37; *p* = 0.0008). In contrast, no significant survival difference was observed between the obese group and the normal weight group (Figure 12). For neurological functional outcome, underweight patients had significantly poorer outcomes compared with the normal weight control group (OR = 0.72, 95% CI 0.55–0.94; *p* = 0.01). No significant difference was observed between the overweight/obese group and the normal weight group (Figure 13).

## 4 Discussion

### 4.1 Ethnicity

Our systematic analysis through meta-analysis, identified a significant racial disparity in hospital discharge survival rates after CPR between Black and White patients in the United States: White patients had a higher survival rate than Black patients (OR = 1.36, 95% CI [1.22, 1.50], *p* < 0.00001). Sensitivity analysis confirmed the good robustness of this result, with no substantial changes observed after the exclusion of any single study. It is worth noting that all included studies in this research were from the United States. This geographic limitation is partly due to the subjective selection of studies comparing Black and White populations in this research, which happened to all be based on U.S. data. Another part is determined by objective research conditions: the United States has a more sophisticated infrastructure in research on race-related health disparities and epidemiological surveillance systems for cardiac arrest, with significantly higher completeness and accessibility of data records than other countries. In contrast, the number of direct comparative studies on Black and White racial cohorts in other countries is relatively small and fails to meet the inclusion criteria.

Further analysis revealed that racial survival disparities within the United States are not uniformly distributed but exhibit significant geographic heterogeneity. Compared with Seattle, the racial survival gap is more prominent in Chicago, while the disparity is relatively moderate in Michigan. This regional differentiation essentially reflects the geographic imbalance in medical resource allocation and socioeconomic structure—key medical factors such as EMS response time, community CPR training coverage, and hospital specialized resource allocation, as well as socioeconomic characteristics like regional income gaps and the degree of racial residential segregation, collectively shape the geographic pattern of racial health inequities. In addition, the significant increase in cardiac arrest-related mortality among Black populations during the COVID-19 pandemic in 2020 further confirms the “magnifying effect” of public health crises on medical inequities: issues such as barriers to accessing health resources and the increased burden of underlying diseases faced by vulnerable groups during crises will exacerbate the already existing racial survival disparities.

The aforementioned racial disparities are not caused by a single factor but result from the interplay of multiple dimensions, which can be analyzed in depth from the following three aspects:

First, structural inequities in the healthcare system are the core root cause. Included studies consistently show that Black patients are more likely to receive treatment in resource-poor medical facilities. For example, a study by Raina et al. (26) pointed out that hospitals in southern U.S. cities serving low-income populations (predominantly Black) lag significantly behind those serving high-income groups in terms of investment in emergency equipment and specialized diagnostic and therapeutic capabilities. A study by Chan et al. (25)further confirmed that after adjusting for hospital characteristics (such as the number of beds and intensive care resources), the survival disparity between races narrowed significantly, directly highlighting the critical impact of uneven institutional resource allocation on outcomes. Such structural disparities are not only reflected in in-hospital care but also permeate the entire healthcare service chain, forming a “vulnerable population → resource-poor institution → poor outcome” vicious cycle.

Second, disparities in prehospital emergency care and pathophysiological characteristics exacerbate outcome differentiation. Relevant studies (52, 53) have pointed out that Black patients have a higher risk of heart disease and cardiovascular diseases, and the incidence of sudden cardiac death is also higher than that of non-Hispanic Whites, which may be related to social and environmental factors. From a pathophysiological perspective, Black patients are more likely to experience non-shockable rhythms such as pulseless electrical activity (PEA) during cardiac arrest. The survival rate of such rhythms is 2–3 times lower than that of shockable rhythms like ventricular fibrillation (VF), constituting an innate prognostic disadvantage (21, 54). From the perspective of prehospital intervention, the implementation rate of bystander CPR is significantly lower in low-income communities with a high concentration of Black people. As a key intervention within the “golden 4 min” after cardiac arrest, the absence of bystander CPR directly reduces the survival probability of patients before they reach the hospital, further widening the outcome gap with White patients.

Third, social determinants of health are important mediating variables. Socioeconomic status (SES) affects the CPR survival chain through multiple pathways: populations with low income and education levels often face problems such as insufficient coverage of emergency medical facilities and lack of chronic disease management, leading to an increased baseline risk of cardiac arrest (19). Meanwhile, economic pressure also limits their access to high-quality prehospital emergency care and post-resuscitation care. A study by Chu et al. (22) provides key evidence: after fully adjusting for SES indicators such as income and education, the survival disparity between Black and White patients largely disappears, indicating that racial disparities are to a large extent a “health manifestation” of socioeconomic inequality. The historical legacies of structural racism further solidify this pattern of inequity.

### 4.2 Past medical history

This study demonstrated that patients without a past medical history had a significantly higher survival rate after CPR than those with a history (pooled OR = 0.51, 95% CI: 0.37–0.70, *p* < 0.0001). These findings suggest that comorbidities may reduce physiological resilience, thereby impairing patients’ ability to withstand cardiac arrest. Although it must be acknowledged that heterogeneity in the definition of past medical history may introduce certain biases, both the forest plot of the meta-analysis and sensitivity analysis confirmed that comorbidities have a significant impact on the survival rate of CPR patients, and the results are robust. The specific mechanisms can be analyzed in depth from the following three aspects:

Firstly, the significant impact of cardiovascular related medical history. Authoritative literature has clearly established that coronary artery disease and cardiovascular risk factors such as diabetes and hypertension significantly increase the prevalence of heart failure and sudden cardiac death, thereby influencing the outcome of CPR in patients with a past medical history. Roth et al. (35)and Bastos et al. (38) identified high cholesterol and markers of coronary artery disease (e.g., low-flow time, lactic acid levels) as independent risk factors. Potential mechanisms include reduced coronary perfusion due to atherosclerosis, myocardial electrical instability, and metabolic acidosis exacerbating post-resuscitation multi-organ dysfunction.

Secondly, non cardiovascular complications also have complex effects. Winther-Jensen et al. (39) pointed out that cancer itself does not directly affect survival, but cancer patients receive targeted temperature management (TTM) less frequently, reflecting a clinical tendency to limit treatment in this population. Dumas et al. (55) used the Charlson Comorbidity Index (CCI) and found that for each 1-point increase in CCI, the risk of in-hospital death increased by 15%, highlighting the synergistic negative impact of multi-organ dysfunction (e.g., chronic kidney disease exacerbates arrhythmia risk through electrolyte disorders).

Thirdly, the influence of pathological and physiological mechanisms. From a pathophysiological perspective, the “organ reserve depletion theory” explains how chronic diseases (e.g., heart failure) affect CPR outcomes: such diseases lead to downregulation of cardiac *β*-receptors, myocardial fibrosis, and reduced responsiveness to adrenergic drugs during CPR. Multiple studies (40, 41) have confirmed that diabetes and blood glucose levels significantly impact survival and recovery after CPR. Metabolic disorders and insulin resistance in diabetic patients further exacerbate post-resuscitation hyperglycemia and oxidative stress-mediated brain injury. In addition, Vukmir et al. (56) observed a negative correlation between the use of antiarrhythmic drugs and survival, which more likely reflects physicians’ medication tendency in high-risk patients (rather than a direct effect of the drugs themselves on outcomes).

In future research and clinical practice, the interaction between “medical record quality” and “pathophysiological burden” warrants further exploration. A study (57) found that patients with “clearly documented medical history” had a higher resuscitation success rate, which may be attributed to comprehensive medical history documentation facilitating informed emergency interventions (including timely identification of reversible factors and adjustment of drug dosages). This conclusion differs from the mainstream view in most studies, so it is necessary to distinguish the independent impacts of “medical history documentation quality” and “pathophysiological load.”

In clinical practice, it is recommended to develop risk stratification tools that integrate the CCI, laboratory markers, and electrocardiographic features to construct predictive models (e.g., the modified CAHP score). Meanwhile, personalized resuscitation strategies should be formulated: for example, prioritizing pneumothorax screening in patients with chronic obstructive pulmonary disease and strengthening blood glucose monitoring in diabetic patients to prevent post-resuscitation hyperosmolarity. Future precision resuscitation medicine needs to establish a closed-loop management system of “history identification-mechanism intervention-prognosis prediction” to achieve precise intervention during the golden period of emergency treatment.

### 4.3 Body mass index

The results of this meta-analysis indicate a nonlinear association between BMI and outcomes after CPR: Underweight patients [BMI < 18.5 kg/m (2)] had significantly lower survival rates and favorable neurological outcomes compared with those with a normal BMI (BMI 18.5–24.9 kg/m^2^, hereinafter referred to as the normal BMI group); in contrast, no statistically significant differences were observed between overweight/obese patients (BMI ≥ 25 kg/m^2^) and the normal BMI group. This finding supports the “obesity paradox”—a hypothesis that traditional metabolic risk factors may exert opposite potential protective effects in critical illness scenarios.

Firstly, an analysis of the mechanism of action of body mass index. Underweight has been confirmed as an independent risk factor for CPR outcomes in multiple studies (51), with the core mechanism possibly related to reduced ischemia tolerance caused by chronic malnutrition. Studies by Lee et al. (45) and Wang et al. (46) both observed that underweight patients had a significantly lower rate of favorable neurological recovery at discharge compared with those with a normal BMI (14.67% vs. 36.04%). Conversely, Testori et al. (48) reported that overweight patients achieved better 6-month survival rates and neurological outcomes. This phenomenon can be explained from a physiological perspective through the dual functions of adipose tissue: On one hand, subcutaneous fat reduces CPR-related complications (e.g., rib fractures) via mechanical buffering. On the other hand, adiponectin secreted by visceral fat inhibits the release of pro-inflammatory molecules such as interleukin-6 (IL-6) and tumor necrosis factor-*α* (TNF-α) after cardiac arrest, alleviating systemic inflammatory responses. However, in cases of severe obesity, the reduction in chest compliance offsets these protective effects—which is consistent with the findings of Shahreyar et al. (58), suggesting a decreased CPR success rate in severely obese patients.

Secondly, the classification of body mass index has certain methodological limitations. As emphasized by Wang et al. (46), the methodological limitations of BMI pose challenges in distinguishing between muscle mass and fat distribution. Their study confirmed that anteroposterior chest diameter (APD) and sagittal abdominal diameter (SAD) are more effective than BMI in predicting resuscitation quality. Meanwhile, significant differences in body composition characteristics exist among populations from different regions: For example, East Asian populations have a higher body fat percentage at the same BMI compared with European and American populations, whereas European and American populations have a relatively higher proportion of muscle mass. Such differences may affect the strength of the association between BMI and CPR outcomes. To a certain extent, BMI is not an ideal indicator for distinguishing muscle mass from obesity, and the observed “paradox” may be a statistical artifact (e.g., confounding by age or disease severity) rather than a true biological protective effect.

Future research should incorporate underweight as an independent risk factor into the resuscitation prognosis evaluation system; utilize new technologies such as bioelectrical impedance analysis (BIA) and computed tomography (CT) to quantify muscle mass and visceral fat content, thereby improving the accuracy of feedback on chest compression effectiveness. Additionally, traditional obesity management strategies need to be re-evaluated in high-risk populations for cardiac arrest—shifting from simple weight loss to optimizing body composition (e.g., improving skeletal muscle mass through resistance training) to enhance physiological reserve. In clinical practice, the “one-size-fits-all” weight management paradigm should be abandoned, and focus should instead be placed on indicators that more accurately reflect patients’ conditions, providing new insights for improving the survival chain of cardiac arrest patients.

## 5 Conclusion

This study systematically explored the differential impacts of three types of individualized factors—race, past medical history, and BMI—on CPR outcomes through systematic review and meta-analysis, clarifying their complex action patterns: Specifically, White patients had a significantly higher CPR survival rate than Black patients; patients without comorbidities showed a substantially increased survival probability compared with those with comorbidities; underweight patients exhibited significantly poorer survival rates and favorable neurological outcomes than patients with a normal weight.

It is important to objectively acknowledge two major methodological limitations of this study: First, the core exposure factors (race, comorbidities, BMI) in CPR outcome research are mostly long-term stable objective characteristics, while the endpoint events (out-of-hospital/in-hospital cardiac arrest) are acute in nature. This has led to the inclusion of predominantly retrospective studies in the present meta-analysis. Although prospective cohort studies can reduce bias, they require long-term follow-up of large populations to accumulate sufficient sample sizes, which is not only time-consuming and resource-intensive but also faces practical challenges such as ethical review restrictions (e.g., inability to actively intervene in factors like race and underlying diseases) and high follow-up loss rates. Therefore, retrospective studies remain the primary method for evidence accumulation in this field to date. Second, for the factor of past medical history, this study did not conduct more detailed subgroup analyses (e.g., stratification by disease type, severity, or stage). The main reason is that some potential subgroups only included 2–3 initial studies with a small total sample size. Forcing stratified analysis would have resulted in insufficient statistical power and questionable result reliability. Thus, the initial study design prioritized verifying the overall effect to ensure the robustness of the core conclusions.

Building on the above limitations, future research should focus on multi-center prospective cohort designs, combined with statistical methods such as propensity score matching, and conduct targeted subgroup analyses for factors like past medical history. This will further verify the applicable scenarios and boundaries of the findings of this study, providing higher-level evidence for the development of personalized CPR strategies. Despite the aforementioned objective constraints related to study design, the core findings of this research still hold significant guiding value for clinical CPR practice, with specific practical recommendations as follows:

### 5.1 Addressing racial disparities in CPR outcomes

Healthcare practitioners should fully recognize the significant impact of race on CPR outcomes and strive to eliminate structural inequities in medical resource allocation. It is recommended to develop prognostic prediction models incorporating SES adjustments, while strengthening CPR skill training and public health education in low-income communities to improve bystander CPR implementation rates. Future research should include data from more low- and middle-income countries (LMICs) to analyze the moderating effect of differences in medical infrastructure on resuscitation outcomes; in addition, prospective cohort studies combining geographic information systems (GIS) and multilevel modeling should be conducted to quantify the interaction between structural factors (e.g., hospital resources) and individual characteristics, providing precise targets for narrowing racial health gaps.

### 5.2 Optimizing Management of Patients with comorbidities

Priority should be given to the standardized management and treatment optimization of comorbidities, with a particular focus on the prevention and control of core comorbidities such as cardiovascular diseases, to enhance patients’ physiological reserve and tolerance to cardiac arrest. It is recommended to establish a full-process closed-loop management system of “medical history identification-mechanism intervention-prognostic prediction.” By integrating multidimensional indicators such as the CCI and laboratory markers, precise intervention throughout the CPR process can be achieved, improving the resuscitation outcomes of patients with comorbidities.

### 5.3 Targeted interventions for BMI-related high-risk populations

Underweight patients should be clearly identified as a high-risk group for CPR. In clinical practice, emphasis should be placed on nutritional support and intervention for potential chronic inflammation in this population, and underweight status should be incorporated into the CPR prognostic evaluation system to optimize risk stratification. Furthermore, public health management strategies related to obesity need to be restructured—shifting from the traditional single goal of weight loss to comprehensive interventions focusing on “optimizing muscle mass and improving overall health status,” such as enhancing skeletal muscle reserve through resistance training to improve tolerance to critical illness.

Future research should further deepen the exploration of the mechanisms by which individualized factors influence CPR outcomes, focusing on the interaction between different factors (e.g., the synergistic effects of race and SES, BMI and comorbidities); simultaneously, promote the optimization and update of relevant clinical guidelines. Through measures such as optimizing resource allocation, developing accurate assessment tools, and strengthening multidisciplinary collaboration, a personalized resuscitation medicine system can be constructed to provide more solid evidence for formulating precise resuscitation strategies. Structural reforms should be advanced to achieve health equity, ultimately improving the overall level of public health and safety.

## Data Availability

The original contributions presented in the study are included in the article/Supplementary material, further inquiries can be directed to the corresponding author.
