# Supplementary material for: Racial disparities, comorbidities, and low body mass index reduce survival after cardiopulmonary resuscitation: a systematic review and meta-analysis
Source: Front Public Health. 2025 Dec 10;13:1663865. doi: 10.3389/fpubh.2025.1663865 (PMC12728053; doi:10.3389/fpubh.2025.1663865)
Supplement: Supplementary file 1 [file Supplementary_file_1.docx]

Supplementary Material

# Search Strategy

The keywords used in the search were PubMed subject terms.

## The search terms for “cardiopulmonary resuscitation” included:

Cardiopulmonary Resuscitation, Resuscitation, Cardiopulmonary, Cardio- Pulmonary Resuscitation, Cardio Pulmonary Resuscitation, Resuscitation, Cardio-Pulmonary, CPR, Mouth-to-Mouth Resuscitation, Mouth to Mouth Resuscitation, Mouth to Mouth Resuscitation, Mouth to Mouth Resuscitation, Mouth to Mouth Resuscitation, Mouth to Mouth Resuscitation. Resuscitation, Mouth-to-Mouth Resuscitations1, Resuscitation, Mouth-to-Mouth, Resuscitations, Mouth-to-Mouth, Code Blue, Basic Cardiac Life Support, Life Support, Basic Cardiac;

## The search terms for “Ethnicity” included :

Population Groups, Group, Population, Population Group;

## The search terms for “Past Medical History” included :

Medical History Taking, History Taking, Medical, Family Medical History, Family Medical Histories, Medical History, Family, Past Medical History, Family, Family History, Medical, Medical Family Histories, Medical Family History, Family History, Medical, Medical Family Histories, Medical Family History, Family History, Health Medical History, Previous, History, Previous Medical, Medical Histories, Previous, Previous Medical Histories;

## The search terms for “Body Mass Index” included :

Body Weight, Body Weights Body Weight, Body Weights, Weight, Body, Weights, Body.

# Database-Specific Search Queries

We reviewed all references listed in the relevant literature and included literature that met the criteria, if any. No language restrictions or methodological filters were used. The search formula followed the PICOS principle, using subject terms in conjunction with free terms and logical operators “AND” and “OR” to form the search formula.

The specific search form is as follows:

## Racial

### Database : Pubmed

("population groups"[MeSH Terms] OR ("population"[All Fields] AND "groups"[All Fields]) OR "population groups"[All Fields] OR ("population groups"[MeSH Terms] OR ("population"[All Fields] AND "groups"[All Fields]) OR "population groups"[All Fields] OR ("group"[All Fields] AND "population"[All Fields]) OR "group population"[All Fields]) OR ("population groups"[MeSH Terms] OR ("population"[All Fields] AND "groups"[All Fields]) OR "population groups"[All Fields] OR ("population"[All Fields] AND "group"[All Fields]) OR "population group"[All Fields])) AND ("cardiopulmonary resuscitation"[MeSH Terms] OR ("cardiopulmonary"[All Fields] AND "resuscitation"[All Fields]) OR "cardiopulmonary resuscitation"[All Fields] OR ("cardiopulmonary resuscitation"[MeSH Terms] OR ("cardiopulmonary"[All Fields] AND "resuscitation"[All Fields]) OR "cardiopulmonary resuscitation"[All Fields] OR ("resuscitation"[All Fields] AND "cardiopulmonary"[All Fields]) OR "resuscitation cardiopulmonary"[All Fields]) OR ("cardiopulmonary resuscitation"[MeSH Terms] OR ("cardiopulmonary"[All Fields] AND "resuscitation"[All Fields]) OR "cardiopulmonary resuscitation"[All Fields] OR ("cardio"[All Fields] AND "pulmonary"[All Fields] AND "resuscitation"[All Fields]) OR "cardio pulmonary resuscitation"[All Fields]) OR ("cardiopulmonary resuscitation"[MeSH Terms] OR ("cardiopulmonary"[All Fields] AND "resuscitation"[All Fields]) OR "cardiopulmonary resuscitation"[All Fields] OR ("cardio"[All Fields] AND "pulmonary"[All Fields] AND "resuscitation"[All Fields]) OR "cardio pulmonary resuscitation"[All Fields]) OR (("resuscitability"[All Fields] OR "resuscitate"[All Fields] OR "resuscitated"[All Fields] OR "resuscitates"[All Fields] OR "resuscitating"[All Fields] OR "resuscitation"[MeSH Terms] OR "resuscitation"[All Fields] OR "resuscitations"[All Fields] OR "resuscitative"[All Fields] OR "resuscitator"[All Fields] OR "resuscitators"[All Fields]) AND "Cardio-Pulmonary"[All Fields]) OR ("cardiopulmonary resuscitation"[MeSH Terms] OR ("cardiopulmonary"[All Fields] AND "resuscitation"[All Fields]) OR "cardiopulmonary resuscitation"[All Fields] OR "cpr"[All Fields]) OR ("cardiopulmonary resuscitation"[MeSH Terms] OR ("cardiopulmonary"[All Fields] AND "resuscitation"[All Fields]) OR "cardiopulmonary resuscitation"[All Fields] OR ("mouth"[All Fields] AND "resuscitation"[All Fields]) OR "mouth to mouth resuscitation"[All Fields]) OR ("cardiopulmonary resuscitation"[MeSH Terms] OR ("cardiopulmonary"[All Fields] AND "resuscitation"[All Fields]) OR "cardiopulmonary resuscitation"[All Fields] OR ("mouth"[All Fields] AND "resuscitation"[All Fields]) OR "mouth to mouth resuscitation"[All Fields]) OR ("cardiopulmonary resuscitation"[MeSH Terms] OR ("cardiopulmonary"[All Fields] AND "resuscitation"[All Fields]) OR "cardiopulmonary resuscitation"[All Fields] OR ("mouth"[All Fields] AND "resuscitations"[All Fields])) OR (("resuscitability"[All Fields] OR "resuscitate"[All Fields] OR "resuscitated"[All Fields] OR "resuscitates"[All Fields] OR "resuscitating"[All Fields] OR "resuscitation"[MeSH Terms] OR "resuscitation"[All Fields] OR "resuscitations"[All Fields] OR "resuscitative"[All Fields] OR "resuscitator"[All Fields] OR "resuscitators"[All Fields]) AND "Mouth-to-Mouth"[All Fields]) OR (("resuscitability"[All Fields] OR "resuscitate"[All Fields] OR "resuscitated"[All Fields] OR "resuscitates"[All Fields] OR "resuscitating"[All Fields] OR "resuscitation"[MeSH Terms] OR "resuscitation"[All Fields] OR "resuscitations"[All Fields] OR "resuscitative"[All Fields] OR "resuscitator"[All Fields] OR "resuscitators"[All Fields]) AND "Mouth-to-Mouth"[All Fields]) OR ("cardiopulmonary resuscitation"[MeSH Terms] OR ("cardiopulmonary"[All Fields] AND "resuscitation"[All Fields]) OR "cardiopulmonary resuscitation"[All Fields] OR ("code"[All Fields] AND "blue"[All Fields]) OR "code blue"[All Fields]) OR ("cardiopulmonary resuscitation"[MeSH Terms] OR ("cardiopulmonary"[All Fields] AND "resuscitation"[All Fields]) OR "cardiopulmonary resuscitation"[All Fields] OR ("basic"[All Fields] AND "cardiac"[All Fields] AND "life"[All Fields] AND "support"[All Fields]) OR "basic cardiac life support"[All Fields]) OR ("cardiopulmonary resuscitation"[MeSH Terms] OR ("cardiopulmonary"[All Fields] AND "resuscitation"[All Fields]) OR "cardiopulmonary resuscitation"[All Fields] OR ("life"[All Fields] AND "support"[All Fields] AND "basic"[All Fields] AND "cardiac"[All Fields]) OR "life support basic cardiac"[All Fields]))

### Database : Web of science

(((Population Groups) OR (Group, Population)) OR (Population Group)) AND ((((((((((((((Cardiopulmonary Resuscitation) OR (Resuscitation, Cardiopulmonary)) OR (Cardio-Pulmonary Resuscitation)) OR (Cardio Pulmonary Resuscitation)) OR (Resuscitation, Cardio-Pulmonary)) OR (CPR)) OR (Mouth-to-Mouth Resuscitation)) OR (Mouth to Mouth Resuscitation)) OR (Mouth-to-Mouth Resuscitations)) OR (Resuscitation, Mouth-to-Mouth)) OR (Resuscitations, Mouth-to-Mouth)) OR (Code Blue)) OR (Basic Cardiac Life Support)) OR (Life Support, Basic Cardiac))

### Database: CNKI

（篇关摘：人种 + 人种学 + 人种差异 + 人种群 + 人种类型 + 种族 + 种族差异(精确)）AND（篇关摘：心肺复苏 + 心肺复苏术 + '心肺复苏(cpr)' + 心脏骤停 + 心搏骤停 + 体外心肺复苏 + 院前急救(精确)）

### Database: Wanfang

主题:(心肺复苏) and 主题:(人种)

### Database: VIP

((((((题名或关键词=心脏骤停 OR 题名或关键词=心搏骤停) OR 题名或关键词=心肺复苏术) OR 题名或关键词=体外心肺复苏) OR 题名或关键词=CPR) OR 题名或关键词=院前急救) AND ((((题名或关键词=人种 OR 题名或关键词=human species) OR 题名或关键词=race) OR 题名或关键词=races of man) OR 题名或关键词=种族))

### 2.1.6 Database: Embase

(‘Cardiopulmonary Resuscitation’:ab,kw,ti OR ‘Resuscitation, Cardiopulmonary’:ab,kw,ti OR ‘Cardio-Pulmonary Resuscitation’:ab,kw,ti OR ‘Cardio Pulmonary Resuscitation’:ab,kw,ti OR ‘Resuscitation, Cardio-Pulmonary’:ab,kw,ti OR ‘CPR’:ab,kw,ti OR ‘Mouth-to-Mouth Resuscitation’:ab,kw,ti OR ‘Mouth to Mouth Resuscitation’:ab,kw,ti OR ‘Mouth-to-Mouth Resuscitations’:ab,kw,ti OR ‘Resuscitation, Mouth-to-Mouth’:ab,kw,ti OR ‘Resuscitations, Mouth-to-Mouth’:ab,kw,ti OR ‘Code Blue’:ab,kw,ti OR ‘Basic Cardiac Life Support’:ab,kw,ti OR ‘Life Support, Basic Cardiac’) AND (‘Population Groups’:ab,kw,ti OR ‘Group, Population’:ab,kw,ti OR ‘Population Group’:ab,kw,ti)

### 2.1.7 Database: Scopus

(("Cardiopulmonary Resuscitation" OR "Resuscitation, Cardiopulmonary" OR "Cardio-Pulmonary Resuscitation" OR "Cardio Pulmonary Resuscitation" OR "Resuscitation, Cardio-Pulmonary" OR "CPR" OR "Mouth-to-Mouth Resuscitation" OR "Mouth to Mouth Resuscitation" OR "Mouth-to-Mouth Resuscitations" OR "Resuscitation, Mouth-to-Mouth" OR "Resuscitations, Mouth-to-Mouth" OR "Code Blue" OR "Basic Cardiac Life Support" OR "Life Support, Basic Cardiac") AND ("Population Groups" OR "Group, Population" OR "Population Group"))

### 2.1.8 Database: Cochrane Library

ID Search Hits

#1 MeSH descriptor: [Cardiopulmonary Resuscitation] explode all trees 1837

#2 (Resuscitation, Cardiopulmonary):ti,ab,kw OR (Cardio-Pulmonary Resuscitation):ti,ab,kw OR (Cardio Pulmonary Resuscitation):ti,ab,kw OR (Resuscitation, Cardio-Pulmonary):ti,ab,kw OR (CPR):ti,ab,kw OR (Mouth-to-Mouth Resuscitation):ti,ab,kw OR (Mouth to Mouth Resuscitation):ti,ab,kw OR (Mouth-to-Mouth Resuscitations):ti,ab,kw OR (Resuscitation, Mouth-to-Mouth):ti,ab,kw OR (Resuscitations, Mouth-to-Mouth):ti,ab,kw OR (Code Blue):ti,ab,kw OR (Basic Cardiac Life Support):ti,ab,kw OR (Life Support, Basic Cardiac):ti,ab,kw 5068

#3 #1 or #2 5118

#4 MeSH descriptor: [Population Groups] explode all trees 13221

#5 (Population Groups):ti,ab,kw OR (Group, Population):ti,ab,kw OR (Population Group):ti,ab,kw 95751

#6 #5 or #4 107050

#7 #3 and #6 282

## Medical history Taking

### Database : Pubmed

("medical history taking"[MeSH Terms] OR ("medical"[All Fields] AND "history"[All Fields] AND "taking"[All Fields]) OR "medical history taking"[All Fields] OR ("medical history taking"[MeSH Terms] OR ("medical"[All Fields] AND "history"[All Fields] AND "taking"[All Fields]) OR "medical history taking"[All Fields] OR ("history"[All Fields] AND "taking"[All Fields] AND "medical"[All Fields]) OR "history taking medical"[All Fields]) OR ("medical history taking"[MeSH Terms] OR ("medical"[All Fields] AND "history"[All Fields] AND "taking"[All Fields]) OR "medical history taking"[All Fields] OR ("family"[All Fields] AND "medical"[All Fields] AND "history"[All Fields]) OR "family medical history"[All Fields]) OR ("medical history taking"[MeSH Terms] OR ("medical"[All Fields] AND "history"[All Fields] AND "taking"[All Fields]) OR "medical history taking"[All Fields] OR ("family"[All Fields] AND "medical"[All Fields] AND "histories"[All Fields]) OR "family medical histories"[All Fields]) OR ("medical history taking"[MeSH Terms] OR ("medical"[All Fields] AND "history"[All Fields] AND "taking"[All Fields]) OR "medical history taking"[All Fields] OR ("medical"[All Fields] AND "history"[All Fields] AND "family"[All Fields]) OR "medical history family"[All Fields]) OR ("medical history taking"[MeSH Terms] OR ("medical"[All Fields] AND "history"[All Fields] AND "taking"[All Fields]) OR "medical history taking"[All Fields] OR ("past"[All Fields] AND "medical"[All Fields] AND "history"[All Fields] AND "family"[All Fields]) OR "past medical history family"[All Fields]) OR ("medical history taking"[MeSH Terms] OR ("medical"[All Fields] AND "history"[All Fields] AND "taking"[All Fields]) OR "medical history taking"[All Fields] OR ("family"[All Fields] AND "history"[All Fields] AND "medical"[All Fields]) OR "family history medical"[All Fields]) OR ("medical history taking"[MeSH Terms] OR ("medical"[All Fields] AND "history"[All Fields] AND "taking"[All Fields]) OR "medical history taking"[All Fields] OR ("medical"[All Fields] AND "family"[All Fields] AND "histories"[All Fields]) OR "medical family histories"[All Fields]) OR ("medical history taking"[MeSH Terms] OR ("medical"[All Fields] AND "history"[All Fields] AND "taking"[All Fields]) OR "medical history taking"[All Fields] OR ("medical"[All Fields] AND "family"[All Fields] AND "history"[All Fields]) OR "medical family history"[All Fields]) OR ("medical history taking"[MeSH Terms] OR ("medical"[All Fields] AND "history"[All Fields] AND "taking"[All Fields]) OR "medical history taking"[All Fields] OR ("family"[All Fields] AND "history"[All Fields] AND "health"[All Fields]) OR "family history health"[All Fields]) OR ("medical history taking"[MeSH Terms] OR ("medical"[All Fields] AND "history"[All Fields] AND "taking"[All Fields]) OR "medical history taking"[All Fields] OR ("health"[All Fields] AND "family"[All Fields] AND "histories"[All Fields])) OR ("medical history taking"[MeSH Terms] OR ("medical"[All Fields] AND "history"[All Fields] AND "taking"[All Fields]) OR "medical history taking"[All Fields] OR ("health"[All Fields] AND "family"[All Fields] AND "history"[All Fields]) OR "health family history"[All Fields]) OR ("medical history taking"[MeSH Terms] OR ("medical"[All Fields] AND "history"[All Fields] AND "taking"[All Fields]) OR "medical history taking"[All Fields] OR ("family"[All Fields] AND "health"[All Fields] AND "history"[All Fields]) OR "family health history"[All Fields]) OR ("medical history taking"[MeSH Terms] OR ("medical"[All Fields] AND "history"[All Fields] AND "taking"[All Fields]) OR "medical history taking"[All Fields] OR ("family"[All Fields] AND "health"[All Fields] AND "histories"[All Fields]) OR "family health histories"[All Fields]) OR ("medical history taking"[MeSH Terms] OR ("medical"[All Fields] AND "history"[All Fields] AND "taking"[All Fields]) OR "medical history taking"[All Fields] OR ("health"[All Fields] AND "history"[All Fields] AND "family"[All Fields]) OR "health history family"[All Fields]) OR ("medical history taking"[MeSH Terms] OR ("medical"[All Fields] AND "history"[All Fields] AND "taking"[All Fields]) OR "medical history taking"[All Fields] OR ("previous"[All Fields] AND "medical"[All Fields] AND "history"[All Fields]) OR "previous medical history"[All Fields]) OR ("medical history taking"[MeSH Terms] OR ("medical"[All Fields] AND "history"[All Fields] AND "taking"[All Fields]) OR "medical history taking"[All Fields] OR ("medical"[All Fields] AND "history"[All Fields] AND "previous"[All Fields]) OR "medical history previous"[All Fields]) OR ("medical history taking"[MeSH Terms] OR ("medical"[All Fields] AND "history"[All Fields] AND "taking"[All Fields]) OR "medical history taking"[All Fields] OR ("history"[All Fields] AND "previous"[All Fields] AND "medical"[All Fields]) OR "history previous medical"[All Fields]) OR ("medical history taking"[MeSH Terms] OR ("medical"[All Fields] AND "history"[All Fields] AND "taking"[All Fields]) OR "medical history taking"[All Fields] OR ("medical"[All Fields] AND "histories"[All Fields] AND "previous"[All Fields])) OR ("medical history taking"[MeSH Terms] OR ("medical"[All Fields] AND "history"[All Fields] AND "taking"[All Fields]) OR "medical history taking"[All Fields] OR ("previous"[All Fields] AND "medical"[All Fields] AND "histories"[All Fields]) OR "previous medical histories"[All Fields])) AND ("cardiopulmonary resuscitation"[MeSH Terms] OR ("cardiopulmonary"[All Fields] AND "resuscitation"[All Fields]) OR "cardiopulmonary resuscitation"[All Fields] OR ("cardiopulmonary resuscitation"[MeSH Terms] OR ("cardiopulmonary"[All Fields] AND "resuscitation"[All Fields]) OR "cardiopulmonary resuscitation"[All Fields] OR ("resuscitation"[All Fields] AND "cardiopulmonary"[All Fields]) OR "resuscitation cardiopulmonary"[All Fields]) OR ("cardiopulmonary resuscitation"[MeSH Terms] OR ("cardiopulmonary"[All Fields] AND "resuscitation"[All Fields]) OR "cardiopulmonary resuscitation"[All Fields] OR ("cardio"[All Fields] AND "pulmonary"[All Fields] AND "resuscitation"[All Fields]) OR "cardio pulmonary resuscitation"[All Fields]) OR ("cardiopulmonary resuscitation"[MeSH Terms] OR ("cardiopulmonary"[All Fields] AND "resuscitation"[All Fields]) OR "cardiopulmonary resuscitation"[All Fields] OR ("cardio"[All Fields] AND "pulmonary"[All Fields] AND "resuscitation"[All Fields]) OR "cardio pulmonary resuscitation"[All Fields]) OR (("resuscitability"[All Fields] OR "resuscitate"[All Fields] OR "resuscitated"[All Fields] OR "resuscitates"[All Fields] OR "resuscitating"[All Fields] OR "resuscitation"[MeSH Terms] OR "resuscitation"[All Fields] OR "resuscitations"[All Fields] OR "resuscitative"[All Fields] OR "resuscitator"[All Fields] OR "resuscitators"[All Fields]) AND "Cardio-Pulmonary"[All Fields]) OR ("cardiopulmonary resuscitation"[MeSH Terms] OR ("cardiopulmonary"[All Fields] AND "resuscitation"[All Fields]) OR "cardiopulmonary resuscitation"[All Fields] OR "cpr"[All Fields]) OR ("cardiopulmonary resuscitation"[MeSH Terms] OR ("cardiopulmonary"[All Fields] AND "resuscitation"[All Fields]) OR "cardiopulmonary resuscitation"[All Fields] OR ("mouth"[All Fields] AND "resuscitation"[All Fields]) OR "mouth to mouth resuscitation"[All Fields]) OR ("cardiopulmonary resuscitation"[MeSH Terms] OR ("cardiopulmonary"[All Fields] AND "resuscitation"[All Fields]) OR "cardiopulmonary resuscitation"[All Fields] OR ("mouth"[All Fields] AND "resuscitation"[All Fields]) OR "mouth to mouth resuscitation"[All Fields]) OR ("cardiopulmonary resuscitation"[MeSH Terms] OR ("cardiopulmonary"[All Fields] AND "resuscitation"[All Fields]) OR "cardiopulmonary resuscitation"[All Fields] OR ("mouth"[All Fields] AND "resuscitations"[All Fields])) OR (("resuscitability"[All Fields] OR "resuscitate"[All Fields] OR "resuscitated"[All Fields] OR "resuscitates"[All Fields] OR "resuscitating"[All Fields] OR "resuscitation"[MeSH Terms] OR "resuscitation"[All Fields] OR "resuscitations"[All Fields] OR "resuscitative"[All Fields] OR "resuscitator"[All Fields] OR "resuscitators"[All Fields]) AND "Mouth-to-Mouth"[All Fields]) OR (("resuscitability"[All Fields] OR "resuscitate"[All Fields] OR "resuscitated"[All Fields] OR "resuscitates"[All Fields] OR "resuscitating"[All Fields] OR "resuscitation"[MeSH Terms] OR "resuscitation"[All Fields] OR "resuscitations"[All Fields] OR "resuscitative"[All Fields] OR "resuscitator"[All Fields] OR "resuscitators"[All Fields]) AND "Mouth-to-Mouth"[All Fields]) OR ("cardiopulmonary resuscitation"[MeSH Terms] OR ("cardiopulmonary"[All Fields] AND "resuscitation"[All Fields]) OR "cardiopulmonary resuscitation"[All Fields] OR ("code"[All Fields] AND "blue"[All Fields]) OR "code blue"[All Fields]) OR ("cardiopulmonary resuscitation"[MeSH Terms] OR ("cardiopulmonary"[All Fields] AND "resuscitation"[All Fields]) OR "cardiopulmonary resuscitation"[All Fields] OR ("basic"[All Fields] AND "cardiac"[All Fields] AND "life"[All Fields] AND "support"[All Fields]) OR "basic cardiac life support"[All Fields]) OR ("cardiopulmonary resuscitation"[MeSH Terms] OR ("cardiopulmonary"[All Fields] AND "resuscitation"[All Fields]) OR "cardiopulmonary resuscitation"[All Fields] OR ("life"[All Fields] AND "support"[All Fields] AND "basic"[All Fields] AND "cardiac"[All Fields]) OR "life support basic cardiac"[All Fields]))

### Database : Web of science

((((((((((((((((((((Medical History Taking) OR (History Taking, Medical)) OR (Family Medical History)) OR (Family Medical Histories)) OR (Medical History, Family)) OR (Past Medical History, Family)) OR (Family History, Medical)) OR (Medical Family Histories)) OR (Medical Family History)) OR (Family History, Health)) OR (Health Family Histories)) OR (Health Family History)) OR (Family Health History)) OR (Family Health Histories)) OR (Health History, Family)) OR (Previous Medical History)) OR (Medical History, Previous)) OR (History, Previous Medical)) OR (Medical Histories, Previous)) OR (Previous Medical Histories)) AND ((((((((((((((Cardiopulmonary Resuscitation) OR (Resuscitation, Cardiopulmonary)) OR (Cardio-Pulmonary Resuscitation)) OR (Cardio Pulmonary Resuscitation)) OR (Resuscitation, Cardio-Pulmonary)) OR (CPR)) OR (Mouth-to-Mouth Resuscitation)) OR (Mouth to Mouth Resuscitation)) OR (Mouth-to-Mouth Resuscitations)) OR (Resuscitation, Mouth-to-Mouth)) OR (Resuscitations, Mouth-to-Mouth)) OR (Code Blue)) OR (Basic Cardiac Life Support)) OR (Life Support, Basic Cardiac))

### Database: CNKI

（主题：既往病史）AND（主题：心肺复苏 + 心肺复苏术 + '心肺复苏(cpr)' + 心脏骤停 + 心搏骤停 + 体外心肺复苏 + 院前急救）

### Database: Wanfang

[主题:(心肺复苏) and 主题:(既往病史)](https://s.wanfangdata.com.cn/advanced-search/paper?q=%E4%B8%BB%E9%A2%98:(%E5%BF%83%E8%82%BA%E5%A4%8D%E8%8B%8F) and %E4%B8%BB%E9%A2%98:(%E6%97%A2%E5%BE%80%E7%97%85%E5%8F%B2)&type=["periodical","thesis","conference"]&chineseEnglishExpand=true&topicExpand=true" \t "https://s.wanfangdata.com.cn/advanced-search/_blank)

### Database: VIP

[((((((题名或关键词=心脏骤停 OR 题名或关键词=心搏骤停) OR 题名或关键词=心肺复苏术) OR 题名或关键词=体外心肺复苏) OR 题名或关键词=CPR) OR 题名或关键词=院前急救) AND (题名或关键词=既往病史 OR 题名或关键词=既往史))](https://qikan.cqvip.com/Qikan/search/index?LngMySearHistoryIdGuid=de9e58c6-ffba-49a8-89b0-dd4670d93832&from=Qikan_Article_History)

### Database: Embase

(‘Cardiopulmonary Resuscitation’:ab,kw,ti OR ‘Resuscitation, Cardiopulmonary’:ab,kw,ti OR ‘Cardio-Pulmonary Resuscitation’:ab,kw,ti OR ‘Cardio Pulmonary Resuscitation’:ab,kw,ti OR ‘Resuscitation, Cardio-Pulmonary’:ab,kw,ti OR ‘CPR’:ab,kw,ti OR ‘Mouth-to-Mouth Resuscitation’:ab,kw,ti OR ‘Mouth to Mouth Resuscitation’:ab,kw,ti OR ‘Mouth-to-Mouth Resuscitations’:ab,kw,ti OR ‘Resuscitation, Mouth-to-Mouth’:ab,kw,ti OR ‘Resuscitations, Mouth-to-Mouth’:ab,kw,ti OR ‘Code Blue’:ab,kw,ti OR ‘Basic Cardiac Life Support’:ab,kw,ti OR ‘Life Support, Basic Cardiac’:ab,kw,ti) AND (‘Medical History Taking’:ab,kw,ti OR ‘History Taking, Medical’:ab,kw,ti OR ‘Family Medical History’:ab,kw,ti OR ‘Family Medical Histories’:ab,kw,ti OR ‘Medical History, Family’:ab,kw,ti OR ‘Past Medical History, Family’:ab,kw,ti OR ‘Family History, Medical’:ab,kw,ti OR ‘Medical Family Histories’:ab,kw,ti OR ‘Medical Family History’:ab,kw,ti OR ‘Family History, Health’:ab,kw,ti OR ‘Health Family Histories’:ab,kw,ti OR ‘Health Family History’:ab,kw,ti OR ‘Family Health History’:ab,kw,ti OR ‘Family Health Histories’:ab,kw,ti OR ‘Health History, Family’:ab,kw,ti OR ‘Previous Medical History’:ab,kw,ti OR ‘Medical History, Previous’:ab,kw,ti OR ‘History, Previous Medical’:ab,kw,ti OR ‘Medical Histories, Previous’:ab,kw,ti OR ‘Previous Medical Histories’:ab,kw,ti)

### Database: Cochrane Library

ID Search Hits

#1 MeSH descriptor: [Cardiopulmonary Resuscitation] explode all trees 1837

#2 (Resuscitation, Cardiopulmonary):ti,ab,kw OR (Cardio-Pulmonary Resuscitation):ti,ab,kw OR (Cardio Pulmonary Resuscitation):ti,ab,kw OR (Resuscitation, Cardio-Pulmonary):ti,ab,kw OR (CPR):ti,ab,kw OR (Mouth-to-Mouth Resuscitation):ti,ab,kw OR (Mouth to Mouth Resuscitation):ti,ab,kw OR (Mouth-to-Mouth Resuscitations):ti,ab,kw OR (Resuscitation, Mouth-to-Mouth):ti,ab,kw OR (Resuscitations, Mouth-to-Mouth):ti,ab,kw OR (Code Blue):ti,ab,kw OR (Basic Cardiac Life Support):ti,ab,kw OR (Life Support, Basic Cardiac):ti,ab,kw 5068

#3 #1 or #2 5118

#12 (Medical History Taking):ti,ab,kw OR (History Taking, Medical):ti,ab,kw OR (Family Medical History):ti,ab,kw OR (Family Medical Histories):ti,ab,kw OR (Medical History, Family):ti,ab,kw OR (Past Medical History, Family):ti,ab,kw OR (Family History, Medical):ti,ab,kw OR (Medical Family Histories):ti,ab,kw OR (Medical Family History):ti,ab,kw OR (Family History, Health):ti,ab,kw OR (Health Family Histories):ti,ab,kw OR (Health Family History):ti,ab,kw OR (Family Health History):ti,ab,kw OR (Family Health Histories):ti,ab,kw OR (Health History, Family):ti,ab,kw OR (Previous Medical History):ti,ab,kw OR (Medical History, Previous):ti,ab,kw OR (History, Previous Medical):ti,ab,kw OR (Medical Histories, Previous):ti,ab,kw OR (Previous Medical Histories):ti,ab,kw 9331

#13 MeSH descriptor: [Medical History Taking] explode all trees 425

#14 #13 or #12 9367

#15 #14 and #3 38

### Database: Scopus

(("Cardiopulmonary Resuscitation" OR "Resuscitation, Cardiopulmonary" OR "Cardio-Pulmonary Resuscitation" OR "Cardio Pulmonary Resuscitation" OR "Resuscitation, Cardio-Pulmonary" OR "CPR" OR "Mouth-to-Mouth Resuscitation" OR "Mouth to Mouth Resuscitation" OR "Mouth-to-Mouth Resuscitations" OR "Resuscitation, Mouth-to-Mouth" OR "Resuscitations, Mouth-to-Mouth" OR "Code Blue" OR "Basic Cardiac Life Support" OR "Life Support, Basic Cardiac") AND ("Medical History Taking" OR "History Taking, Medical" OR "Family Medical History" OR "Family Medical Histories" OR "Medical History, Family" OR "Past Medical History, Family" OR "Family History, Medical" OR "Medical Family Histories" OR "Medical Family History" OR "Family History, Health" OR "Health Family Histories" OR "Health Family History" OR "Family Health History" OR "Family Health Histories" OR "Health History, Family" OR "Previous Medical History" OR "Medical History, Previous" OR "History, Previous Medical" OR "Medical Histories, Previous" OR "Previous Medical Histories"))

## Body Weight

### Database : Pubmed

("body weight"[MeSH Terms] OR ("body"[All Fields] AND "weight"[All Fields]) OR "body weight"[All Fields] OR ("body weight"[MeSH Terms] OR ("body"[All Fields] AND "weight"[All Fields]) OR "body weight"[All Fields] OR ("body"[All Fields] AND "weights"[All Fields]) OR "body weights"[All Fields]) OR ("body weight"[MeSH Terms] OR ("body"[All Fields] AND "weight"[All Fields]) OR "body weight"[All Fields] OR ("weight"[All Fields] AND "body"[All Fields]) OR "weight body"[All Fields]) OR ("body weight"[MeSH Terms] OR ("body"[All Fields] AND "weight"[All Fields]) OR "body weight"[All Fields] OR ("weights"[All Fields] AND "body"[All Fields]) OR "weights body"[All Fields])) AND ("cardiopulmonary resuscitation"[MeSH Terms] OR ("cardiopulmonary"[All Fields] AND "resuscitation"[All Fields]) OR "cardiopulmonary resuscitation"[All Fields] OR ("cardiopulmonary resuscitation"[MeSH Terms] OR ("cardiopulmonary"[All Fields] AND "resuscitation"[All Fields]) OR "cardiopulmonary resuscitation"[All Fields] OR ("resuscitation"[All Fields] AND "cardiopulmonary"[All Fields]) OR "resuscitation cardiopulmonary"[All Fields]) OR ("cardiopulmonary resuscitation"[MeSH Terms] OR ("cardiopulmonary"[All Fields] AND "resuscitation"[All Fields]) OR "cardiopulmonary resuscitation"[All Fields] OR ("cardio"[All Fields] AND "pulmonary"[All Fields] AND "resuscitation"[All Fields]) OR "cardio pulmonary resuscitation"[All Fields]) OR ("cardiopulmonary resuscitation"[MeSH Terms] OR ("cardiopulmonary"[All Fields] AND "resuscitation"[All Fields]) OR "cardiopulmonary resuscitation"[All Fields] OR ("cardio"[All Fields] AND "pulmonary"[All Fields] AND "resuscitation"[All Fields]) OR "cardio pulmonary resuscitation"[All Fields]) OR (("resuscitability"[All Fields] OR "resuscitate"[All Fields] OR "resuscitated"[All Fields] OR "resuscitates"[All Fields] OR "resuscitating"[All Fields] OR "resuscitation"[MeSH Terms] OR "resuscitation"[All Fields] OR "resuscitations"[All Fields] OR "resuscitative"[All Fields] OR "resuscitator"[All Fields] OR "resuscitators"[All Fields]) AND "Cardio-Pulmonary"[All Fields]) OR ("cardiopulmonary resuscitation"[MeSH Terms] OR ("cardiopulmonary"[All Fields] AND "resuscitation"[All Fields]) OR "cardiopulmonary resuscitation"[All Fields] OR "cpr"[All Fields]) OR ("cardiopulmonary resuscitation"[MeSH Terms] OR ("cardiopulmonary"[All Fields] AND "resuscitation"[All Fields]) OR "cardiopulmonary resuscitation"[All Fields] OR ("mouth"[All Fields] AND "resuscitation"[All Fields]) OR "mouth to mouth resuscitation"[All Fields]) OR ("cardiopulmonary resuscitation"[MeSH Terms] OR ("cardiopulmonary"[All Fields] AND "resuscitation"[All Fields]) OR "cardiopulmonary resuscitation"[All Fields] OR ("mouth"[All Fields] AND "resuscitation"[All Fields]) OR "mouth to mouth resuscitation"[All Fields]) OR ("cardiopulmonary resuscitation"[MeSH Terms] OR ("cardiopulmonary"[All Fields] AND "resuscitation"[All Fields]) OR "cardiopulmonary resuscitation"[All Fields] OR ("mouth"[All Fields] AND "resuscitations"[All Fields])) OR (("resuscitability"[All Fields] OR "resuscitate"[All Fields] OR "resuscitated"[All Fields] OR "resuscitates"[All Fields] OR "resuscitating"[All Fields] OR "resuscitation"[MeSH Terms] OR "resuscitation"[All Fields] OR "resuscitations"[All Fields] OR "resuscitative"[All Fields] OR "resuscitator"[All Fields] OR "resuscitators"[All Fields]) AND "Mouth-to-Mouth"[All Fields]) OR (("resuscitability"[All Fields] OR "resuscitate"[All Fields] OR "resuscitated"[All Fields] OR "resuscitates"[All Fields] OR "resuscitating"[All Fields] OR "resuscitation"[MeSH Terms] OR "resuscitation"[All Fields] OR "resuscitations"[All Fields] OR "resuscitative"[All Fields] OR "resuscitator"[All Fields] OR "resuscitators"[All Fields]) AND "Mouth-to-Mouth"[All Fields]) OR ("cardiopulmonary resuscitation"[MeSH Terms] OR ("cardiopulmonary"[All Fields] AND "resuscitation"[All Fields]) OR "cardiopulmonary resuscitation"[All Fields] OR ("code"[All Fields] AND "blue"[All Fields]) OR "code blue"[All Fields]) OR ("cardiopulmonary resuscitation"[MeSH Terms] OR ("cardiopulmonary"[All Fields] AND "resuscitation"[All Fields]) OR "cardiopulmonary resuscitation"[All Fields] OR ("basic"[All Fields] AND "cardiac"[All Fields] AND "life"[All Fields] AND "support"[All Fields]) OR "basic cardiac life support"[All Fields]) OR ("cardiopulmonary resuscitation"[MeSH Terms] OR ("cardiopulmonary"[All Fields] AND "resuscitation"[All Fields]) OR "cardiopulmonary resuscitation"[All Fields] OR ("life"[All Fields] AND "support"[All Fields] AND "basic"[All Fields] AND "cardiac"[All Fields]) OR "life support basic cardiac"[All Fields]))

### Database : Web of science

((((Body Weight) OR (Body Weights)) OR (Weight, Body)) OR (Weights, Body)) AND ((((((((((((((Cardiopulmonary Resuscitation) OR (Resuscitation, Cardiopulmonary)) OR (Cardio-Pulmonary Resuscitation)) OR (Cardio Pulmonary Resuscitation)) OR (Resuscitation, Cardio-Pulmonary)) OR (CPR)) OR (Mouth-to-Mouth Resuscitation)) OR (Mouth to Mouth Resuscitation)) OR (Mouth-to-Mouth Resuscitations)) OR (Resuscitation, Mouth-to-Mouth)) OR (Resuscitations, Mouth-to-Mouth)) OR (Code Blue)) OR (Basic Cardiac Life Support)) OR (Life Support, Basic Cardiac))

### Database: CNKI

（主题：体重 + 体重指数 + '体重指数(bmi)' + 体重差异）AND（主题：心肺复苏 + 心肺复苏术 + '心肺复苏(cpr)' + 心脏骤停 + 心搏骤停 + 体外心肺复苏 + 院前急救

### Database: Wanfang

主题:(心肺复苏) and 主题:(体重) and 主题:(对照)

### Database: VIP

(((题名或关键词=心肺复苏 OR 题名或关键词=心肺复苏术) OR 题名或关键词=胸外按压) AND (((((((题名或关键词=体重 OR 题名或关键词=avoirdupois) OR 题名或关键词=body weight) OR 题名或关键词=bodyweight) OR 题名或关键词=live body weight) OR 题名或关键词=live weight) OR 题名或关键词=weight) OR 题名或关键词=体质量))

### Database: Scopus

(("Cardiopulmonary Resuscitation" OR "Resuscitation, Cardiopulmonary" OR "Cardio-Pulmonary Resuscitation" OR "Cardio Pulmonary Resuscitation" OR "Resuscitation, Cardio-Pulmonary" OR "CPR" OR "Mouth-to-Mouth Resuscitation" OR "Mouth to Mouth Resuscitation" OR "Mouth-to-Mouth Resuscitations" OR "Resuscitation, Mouth-to-Mouth" OR "Resuscitations, Mouth-to-Mouth" OR "Code Blue" OR "Basic Cardiac Life Support" OR "Life Support, Basic Cardiac") AND ("Body Weight" OR "Body Weights" OR "Weight, Body" OR "Weights, Body"))

### Database: Embase

(‘Cardiopulmonary Resuscitation’:ab,kw,ti OR ‘Resuscitation, Cardiopulmonary’:ab,kw,ti OR ‘Cardio-Pulmonary Resuscitation’:ab,kw,ti OR ‘Cardio Pulmonary Resuscitation’:ab,kw,ti OR ‘Resuscitation, Cardio-Pulmonary’:ab,kw,ti OR ‘CPR’:ab,kw,ti OR ‘Mouth-to-Mouth Resuscitation’:ab,kw,ti OR ‘Mouth to Mouth Resuscitation’:ab,kw,ti OR ‘Mouth-to-Mouth Resuscitations’:ab,kw,ti OR ‘Resuscitation, Mouth-to-Mouth’:ab,kw,ti OR ‘Resuscitations, Mouth-to-Mouth’:ab,kw,ti OR ‘Code Blue’:ab,kw,ti OR ‘Basic Cardiac Life Support’:ab,kw,ti OR ‘Life Support, Basic Cardiac’) AND (‘Population Groups’:ab,kw,ti OR ‘Group, Population’:ab,kw,ti OR ‘Population Group’:ab,kw,ti)

### Database: Cochrane Library

ID Search Hits

#1 MeSH descriptor: [Cardiopulmonary Resuscitation] explode all trees 1837

#2 (Resuscitation, Cardiopulmonary):ti,ab,kw OR (Cardio-Pulmonary Resuscitation):ti,ab,kw OR (Cardio Pulmonary Resuscitation):ti,ab,kw OR (Resuscitation, Cardio-Pulmonary):ti,ab,kw OR (CPR):ti,ab,kw OR (Mouth-to-Mouth Resuscitation):ti,ab,kw OR (Mouth to Mouth Resuscitation):ti,ab,kw OR (Mouth-to-Mouth Resuscitations):ti,ab,kw OR (Resuscitation, Mouth-to-Mouth):ti,ab,kw OR (Resuscitations, Mouth-to-Mouth):ti,ab,kw OR (Code Blue):ti,ab,kw OR (Basic Cardiac Life Support):ti,ab,kw OR (Life Support, Basic Cardiac):ti,ab,kw 5068

#3 #1 or #2 5118

#4 MeSH descriptor: [Population Groups] explode all trees 13221

#5 (Population Groups):ti,ab,kw OR (Group, Population):ti,ab,kw OR (Population Group):ti,ab,kw 95751

#6 #5 or #4 107050

#7 #3 and #6 282

# Risk of bias in studies: Newcastle-Ottawa Scale (NOS) Scoring Criteria

The Newcastle-Ottawa Scale (NOS) is a standardized tool for assessing the quality of non-randomized studies in meta-analyses. Its scoring system comprises three domains:

1. Selection (0-4 stars): Evaluates representativeness of exposed/unexposed cohorts, selection of non-exposed cohort, exposure ascertainment, and demonstration that outcomes were not present at baseline.
2. Comparability (0-2 stars): Assesses control for confounding factors through study design or analysis.

3.Outcome/Exposure (0-3 stars): Examines outcome assessment method, adequacy of follow-up duration, and completeness of follow-up.

Each criterion is allocated a maximum of one star (*), except comparability which allows two stars. Total scores range from 0 to 9, with studies categorized as follows: low risk of bias (7-9 stars), moderate risk (5-6 stars), or high risk (0-4 stars). The complete scoring rubric, including domain-specific thresholds for star allocation, is presented in Table 1-3.

Table 1: Body Weight

| Study | Selection | | | | Comparability | Outcome | | | Total |
| --- | --- | --- | --- | --- | --- | --- | --- | --- | --- |
|  | Representativeness of the exposed cohort | Selection of the non-exposed cohort | Ascertainment of exposure | Demonstration that outcome was not present at baseline | Comparability of cohorts | Assessment of outcome | Adequacy of follow-up duration | Completeness of follow-up |  |
| Jain2010 | 1 | 1 | 1 | 1 | 2 | 1 | 1 | 1 | 9 |
| Testori2011 | 1 | 1 | 1 | 1 | 1 | 1 | 1 | 1 | 8 |
| Aoki2018 | 1 | 1 | 1 | 1 | 2 | 1 | 1 | 1 | 9 |
| wang2018 | 1 | 1 | 1 | 1 | 2 | 1 | 1 | 1 | 9 |
| Aoki2024 | 1 | 1 | 1 | 1 | 2 | 1 | 1 | 1 | 9 |
| lee2021 | 1 | 1 | 1 | 1 | 1 | 1 | 1 | - | 7 |
| Ikemura2025 | 1 | 1 | 1 | 1 | 2 | 1 | 1 | 1 | 9 |

Table 2: Medical History

| Study | Selection | | | | Comparability | Outcome | | | | Total |
| --- | --- | --- | --- | --- | --- | --- | --- | --- | --- | --- |
|  | Representativeness of the exposed cohort | Selection of the non-exposed cohort | Ascertainment of exposure | Demonstration that outcome was not present at baseline | Comparability of cohorts | Assessment of outcome | | Adequacy of follow-up duration | Completeness of follow-up |  |
| Bastos2019 | 1 | - | 1 | 1 | 1 | 1 | 1 | | 1 | 8 |
| Roth2000 | 1 | - | 1 | 1 | 1 | 1 | 1 | | - | 7 |
| Winther2020 | 1 | 1 | 1 | 1 | 1 | 1 | 1 | | - | 8 |
| ZhaoLi2007 | 1 | 1 | 1 | 1 | 2 | 1 | 1 | | - | 8 |
| ChenZejiang2015 | 1 | 1 | 1 | 1 | 2 | 1 | 1 | | - | 8 |
| Nehme2016 | 1 | 1 | 1 | 1 | 1 | 1 | 1 | | 1 | 8 |
| Movahedi2017 | 0 | 1 | 1 | 1 | 1 | 1 | 1 | | 1 | 7 |
| Hagglund2023 | 1 | 1 | 1 | 1 | 2 | 1 | 1 | | 1 | 9 |
| Saeed2016 | 1 | 1 | 1 | 1 | 2 | 1 | 1 | | 1 | 9 |

Table 3: Racial

| Study | Selection | | | | Comparability | Outcome | | | Total |
| --- | --- | --- | --- | --- | --- | --- | --- | --- | --- |
|  | Representativeness of the exposed cohort | Selection of the non-exposed cohort | Ascertainment of exposure | Demonstration that outcome was not present at baseline | Comparability of cohorts | Assessment of outcome | Adequacy of follow-up duration | Completeness of follow-up |  |
| Becker1993 | 1 | 1 | 1 | 1 | 2 | 1 | - | - | 7 |
| chan2009 | 1 | 1 | 1 | 1 | 2 | 1 | - | - | 7 |
| chu1998 | 1 | 1 | 1 | 1 | 2 | 1 | - | - | 7 |
| cowie1993 | 1 | 1 | 1 | 1 | 1 | 1 | - | 1 | 7 |
| galea2007 | 1 | 1 | 1 | 1 | 1 | 1 | 1 | 1 | 8 |
| huebinger2022 | 1 | 1 | 1 | 1 | 1 | 1 | 1 | 1 | 8 |
| joseph2017 | 1 | 1 | 1 | 1 | 1 | 1 | - | - | 6 |
| Raina2011 | 1 | 1 | 1 | 1 | 1 | 1 | - | - | 6 |
| sayegh1999 | 1 | 1 | 1 | 1 | 1 | 1 | - | - | 6 |
| Brooks2021 | 1 | 1 | 1 | 1 | 2 | 1 | 1 | 1 | 9 |
| Rabia2015 | 1 | 1 | 1 | 1 | 2 | 1 | 1 | 1 | 9 |
| Shah2009 | 1 | 1 | 1 | 1 | 1 | 1 | 1 | 1 | 8 |
